# Supplementary material for: Cardiovascular risk and cognitive performance: A population-based cross-sectional study (NEDICES2-RISK)
Source: PLoS One. 2026 Mar 25;21(3):e0345086. doi: 10.1371/journal.pone.0345086 (PMC13016341; doi:10.1371/journal.pone.0345086)

S1 Figure. Directed Acyclic Graph (DAG) created with DAGitty v3.1 to identify paths of confusion between exposure (cardiovascular risk) and outcome (cognitive performance).

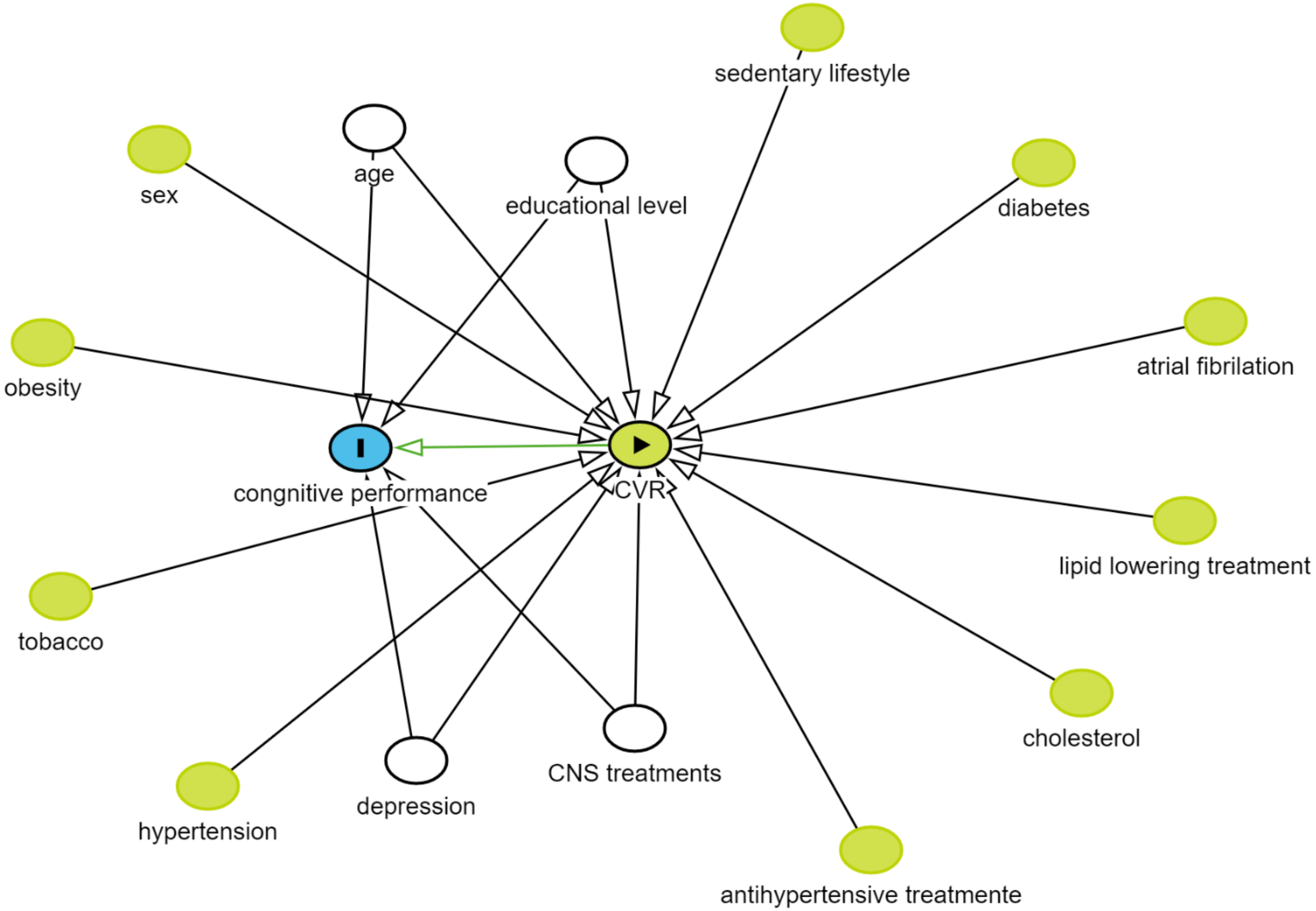

Supplement: S1 Fig — (PDF) [file pone.0345086.s001.pdf]
