# Supplementary material for: Cardiovascular risk and cognitive performance: A population-based cross-sectional study (NEDICES2-RISK)
Source: PLoS One. 2026 Mar 25;21(3):e0345086. doi: 10.1371/journal.pone.0345086 (PMC13016341; doi:10.1371/journal.pone.0345086)
Supplement: S1 Table — (PDF) [file pone.0345086.s002.pdf]

S1 Table. Baseline characteristics and outcomes of cognitive performance tests by the presence or absence of cardiovascular events (stroke, ischemic heart disease, peripheral arterial disease)

Variables	Women				Men			
	**No CVE (n=486)**	**CVE (n=21)**	**Overall (N=507)**	***p***	**No CVE (n=382)**	**CVE (n=73)**	**Overall (N=455)**	***p***
**Age**[1]	67.0 [62.0–71.0]	71.0 [68.0–73.0]	67.0 [62.0–71.0]	0.005[a]	67.0 [62.0–71.0]	69.0 [64.0–73.0]	67.0 [62.0–71.0]	0.028[a]
**Education level**[2]								
No education-Primary	315 (65.5)	15 (71.4)	330 (65.7)	0.744[b]	218 (57.8)	39 (54.9)	257 (57.4)	0.748[b]
Secondary-Superior	166 (34.5)	6 (28.6)	172 (34.3)		159 (42.2)	32 (45.1)	191 (42.6)	
**Smoking**[2]								
Non-smoker	316 (65.4)	12 (63.2)	328 (65.3)	0.937[c]	106 (27.9)	7 (9.6)	113 (24.9)	0.002[b]
Smoker	59 (12.2)	2 (10.5)	61 (12.2)		56 (14.7)	18 (24.7)	74 (16.3)	
Ex-smoker	108 (22.4)	5 (26.3)	113 (22.5)		218 (57.4)	48 (65.8)	266 (58.7)	
**Sedentarism**[2]	322 (66.7)	20 (95.2)	342 (67.9)	0.004[c]	233 (61.6)	55 (75.3)	288 (63.9)	0.036[b]
**Hypertension**[2]	219 (45.1)	15 (71.4)	234 (46.2)	0.032[b]	175 (45.8)	52 (71.2)	227 (49.9)	<0.001[b]
**Diabetes Mellitus**[2]	62 (12.8)	6 (28.6)	68 (13.4)	0.079[b]	84 (22.0)	32 (43.8)	116 (25.5)	<0.001[b]
**Dyslipidemia**[2]	244 (50.2)	17 (81.0)	261 (51.5)	0.007[c]	171 (44.8)	65 (89.0)	236 (51.9)	<0.001[b]
**Atrial fibrillation**[2]	12 (2.5)	1 (4.8)	13 (2.6)	0.427[c]	24 (6.3)	11 (15.1)	35 (7.7)	0.019[b]
**Depression**[2]	87 (17.9)	5 (23.8)	92 (18.1)	0.690[b]	33 (8.6)	3 (4.1)	36 (7.9)	0.281[c]
**CNS treatment**[1]	145 (29.8)	7 (33.3)	152 (30.0)	0.921[b]	63 (16.5)	19 (26.0)	82 (18.0)	0.076[b]
**BMI**[1]	27.6 [24.9–30.6]	27.2 [22.6–29.7]	27.6 [24.8–30.5]	0.288[a]	28.6 [26.6–30.7]	28.9 [26.5–31.5]	28.7 [26.6–30.8]	0.447[a]
**SBP**[1]	130.0 [120.0–140.0]	130.0 [120.0–140.0]	130.0 [120.0–140.0]	0.978[a]	132.0[121.0–140.0]	135.0 [120.0–146.0]	132.0 [120.5–142.0]	0.440[a]
**DBP**[1]	75.0 [70.0–80.0]	70.0 [64.0–71.0]	75.0 [70.0–80.0]	<0.001[a]	78.0 [70.0–85.0]	73.0 [68.0–80.0]	77.0 [70.0–85.0]	0.002[a]
**Total cholesterol**[1]	208.0 [184.0–231.0]	169.0 [159.0–200.0]	207.5 [182.0–230.8]	<0.001[a]	192.0 [167.0–215.0]	152.5 [123.8–181.3]	186.0 [160.0–212.0]	<0.001[a]
**HDL–c**[1]	57.0 [49.0–67.0]	60.0 [50.0–65.0]	57.0 [49.0–67.0]	0.654[a]	49.0 [40.0–57.0]	43.0 [36.0–51.3]	48.0 [40.0–56.3]	0.002[a]
**Cognitive performance test outcomes**[1]								
**Global cognition**								
MMSE-37	33.0 [30.0–35.0]	31.0 [27.0–34.0]	33.0 [30.0–35.0]	0.089[a]	34.0 [31.0–36.0]	34.0 [32.0–36.0]	34.0 [31.0–36.0]	0.944[a]
**Memory**								
Immediate Memory	5.0 [4.0–6.0]	5.0 [4.0–5.0]	5.0 [4.0–6.0]	0.439[a]	5.0 [4.0–6.0]	5.0 [4.0–6.0]	5.0 [4.0–6.0]	0.089[a]
Delayed Recall	5.0 [4.0–6.0]	4.0 [4.0–5.0]	5.0 [4.0–6.0]	0.036[a]	5.0 [4.0–6.0]	5.0 [4.0–6.0]	5.0 [4.0–6.0]	0.156[a]
**Premorbid intelligence**								
Word Accentuation	25.0 [21.0–27.0]	25.0 [21.0–27.0]	25.0 [21.0–27.0]	0.821[a]	25.0 [21.0–28.0]	26.0 [23.0–28.0]	25.0 [21.0–28.0]	0.266[a]

**Verbal fluency**								
Verbal fluency	17.0 [14.0–21.0]	15.0 [10.0–19.0]	17.0 [14.0–21.0]	0.082[a]	18.0 [15.0–22.0]	17.0 [14.0–21.0]	18.0 [14.5–22.0]	0.115[a]
**Visuoconstruction**								
Clock Drawing	4.0 [3.0–4.0]	4.0 [3.0–4.0]	4.0 [3.0–4.0]	0.237[a]	4.0 [3.0–4.0]	4.0 [3.0–4.0]	4.0 [3.0–4.0]	0.472[a]
**Attention and psychomotor speed**								
TMTA-1	60.0 [45.0–81.0]	75.0 [48.0–115.0]	60.0 [45.0–81.5]	0.101[a]	56.0 [41.0–78.0]	52.5 [42.0–65.8]	55.0 [41.0–78.0]	0.457[a]
TMTA-2	68.0 [50.0–103.0]	82.0 [47.0–145.0]	69.0 [50.0–103.8]	0.189[a]	63.0 [43.5–100.0]	61.5 [47.0–85.8]	63.0 [44.0–97.0]	0.527[a]
TMTA-Errors 1	0.0 [0.0–1.0]	1.0 [0.0–3.0]	0.0 [0.0–1.0]	0.002[a]	0.0 [0.0–0.0]	0.0 [0.0–1.0]	0.0 [0.0–0.0]	0.493[a]
TMTA-Errors 2	0.0 [0.0–2.0]	1.0 [1.0–3.0]	1.0 [0.0–2.0]	0.011[a]	0.0 [0.0–1.0]	0.0 [0.0–1.0]	0.0 [0.0–1.0]	0.165[a]

CVE: Cardiovascular event; BMI: Body mass index; SBP: Systolic blood pressure (mmHg); DBP: Diastolic blood pressure (mmHg); HDL-c: High Density Lipoprotein cholesterol; CNS treatment: Treatments that modulate the central nervous system; MMSE–37: Minimental State Examination 37-item version; TMTA: Trail Making Test series A (seconds); 1: median [Q1–Q3]; 2: n (%); a: Mann-Whitney U test; b: Chi-squared test; c: Fisher's test.
