# Supplementary material for: Cardiovascular risk and cognitive performance: A population-based cross-sectional study (NEDICES2-RISK)
Source: PLoS One. 2026 Mar 25;21(3):e0345086. doi: 10.1371/journal.pone.0345086 (PMC13016341; doi:10.1371/journal.pone.0345086)
Supplement: S2 Table — Comparison between participants with the lowest score in the MMSE-37 and the rest. (PDF) [file pone.0345086.s003.pdf]

S2 Table. Baseline characteristics of the sample and cardiovascular risk. Comparison between participants with the lowest score in the MMSE-37 and the rest.

	**Women**				**Men**			
	**≤P25 (n=182)**	**>P25 (n=325)**	**Overall (N=507)**	***p***	**≤P25 (n=115)**	**>P25 (n=340)**	**Overall (N=455)**	***p***
**Age[1]**	70.0 [65.0–72.0]	66.0 [61.0–70.0]	67.0 [62.0–71.0]	<0.001[a]	70.0 [65.0–73.0]	66.0 [61.0–70.0]	67.0 [62.0–71.0]	<0.001[a]
**Education level[2]**								
No education-Primary	152 (85.4)	178 (54.9)	330 (65.7)	<0.001[b]	96 (85.7)	161 (47.9)	257 (57.4)	<0.001[b]
Secondary-Superior	26 (14.6)	146 (45.1)	172 (34.3)		16 (14.3)	175 (52.1)	191 (42.6)	
**Smoking[2]**								
Non-smoker	135 (75.4)	193 (59.8)	328 (65.3)	0.002[b]	32 (28.3)	81 (23.8)	113 (24.9)	0.375[b]
Smoker	16 (8.9)	45 (13.9)	61 (12.2)		21 (18.6)	53 (15.6)	74 (16.3)	
Ex-smoker	28 (15.6)	85 (26.3)	113 (22.5)		60 (53.1)	206 (60.6)	266 (58.7)	
**Sedentary lifestyle[2]**	141 (77.9)	201 (62.2)	342 (67.9)	<0.001[b]	85 (75.2)	203 (60.1)	288 (63.9)	0.005[b]
**Hypertension[2]**	97 (53.3)	137 (42.2)	234 (46.2)	0.020[b]	63 (54.8)	164 (48.2)	227 (49.9)	0.269[b]
**Diabetes Mellitus[2]**	34 (18.7)	34 (10.5)	68 (13.4)	0.014[b]	35 (30.4)	81 (23.8)	116 (25.5)	0.200[b]
**Dyslipidemia[2]**	105 (57.7)	156 (48.0)	261 (51.5)	0.045[b]	51 (44.3)	185 (54.4)	236 (51.9)	0.079[b]
**Atrial fibrillation[2]**	4 (2.2)	9 (2.8)	13 (2.6)	0.778[c]	9 (7.8)	26 (7.6)	35 (7.7)	1.000[b]
**Depression[2]**	36 (19.8)	56 (17.2)	92 (18.1)	0.552[b]	12 (10.4)	24 (7.1)	36 (7.9)	0.337[b]
**CNS treatment[1]**	60 (33.0)	92 (28.3)	152 (30.0)	0.319[b]	26 (22.6)	56 (16.5)	82 (18.0)	0.180[b]
**BMI[1]**	28.1 [25.2–31.2]	27.0 [24.7–30.4]	27.6 [24.8–30.5]	0.054[a]	29.4 [27.2–31.1]	28.6 [26.5–30.5]	28.7 [26.6–30.8]	0.156[a]
**SBP[1]**	131.0 [120.0–140.0]	130.0 [120.0–140.0]	130.0 [120.0–140.0]	0.041[a]	132.0 [120.0–143.5]	132.0 [121.0–140.0]	132.0 [120.5–142.0]	0.879[a]
**DBP[1]**	75.0 [70.0–80.0]	75.0 [70.0–80.0]	75.0 [70.0–80.0]	0.664[a]	76.0 [70.0–84.5]	77.5 [70.0–85.0]	77.0 [70.0–85.0]	0.272[a]
**Total cholesterol[1]**	197.0 [174.0–226.8]	211.0 [187.0–233.3]	207.0 [182.0–230.8]	<0.001[a]	186.0 [163.0–209.8]	186.0 [158.5–213.0]	186.0 [160.0–212.3]	0.856[a]
**HDL–c[1]**	56.0 [47.0–64.0]	58.0 [50.0–69.0]	57.0 [49.0–67.0]	0.031[a]	47.0 [38.0–56.8]	48.0 [41.0–56.0]	48.0 [40.0–56.3]	0.318[a]
**REGICOR[2]**								
Low CVR	117 (77.0)	242 (81.8)	359 (80.1)	0.486[b]	27 (32.5)	121 (45.8)	148 (42.7)	0.011[b]
Moderate CVR	33 (21.7)	51 (17.2)	84 (18.8)		37 (44.6)	114 (43.2)	151 (43.5)	
High CVR	2 (1.3)	3 (1.0)	5 (1.1)		19 (22.9)	29 (11.0)	48 (13.8)	
**FRESCO[2]**								
Low CVR	49 (50.5)	135 (65.5)	184 (60.7)	0.032[b]	10 (14.7)	58 (31.5)	68 (27.0)	0.017[b]
Moderate CVR	38 (39.2)	60 (29.1)	98 (32.3)		32 (47.1)	79 (42.9)	111 (44.0)	
High CVR	10 (10.3)	11 (5.3)	21 (6.9)		26 (38.2)	47 (25.5)	73 (29.0)	

MMSE–37: Minimental State Examination 37 item-version; BMI: Body mass index; SBP: Systolic blood pressure (mmHg); DBP: Diastolic blood pressure (mmHg); HDL-c: High Density Lipoprotein cholesterol; CNS treatment: treatments that modulate the central nervous system; CVR: Cardiovascular risk. 1: median [Q1–Q3]; 2: n (%); a: Mann-Whitney *U test*; b: Chi-squared test; c: Fisher's test.
