# Supplementary material for: Cardiovascular risk and cognitive performance: A population-based cross-sectional study (NEDICES2-RISK)
Source: PLoS One. 2026 Mar 25;21(3):e0345086. doi: 10.1371/journal.pone.0345086 (PMC13016341; doi:10.1371/journal.pone.0345086)
Supplement: S3 Table — Comparison between participants with the worst score in the Inmediate recall test and the rest. (PDF) [file pone.0345086.s004.pdf]

S3 Table. Baseline characteristics of the sample and cardiovascular risk. Comparison between participants with the worst score in the Immediate Recall test and the rest.

	Women				Men			
	**≤P25 (n=171)**	**>P25 (n=335)**	**Overall (N=506)**	***p***	**≤P25 (n=185)**	**>P25 (n=269)**	**Overall (N=454)**	***p***
**Age[1]**	69.0 [64.0–72.0]	66.0 [61.0–71.0]	67.0 [62.0–71.0]	<0.001[a]	69.0 [65.0–73.0]	65.0 [61.0–70.0]	67.0 [62.0–71.0]	<0.001[a]
**Education level[2]**								
No education-Primary	117 (68.8)	212 (64.0)	329 (65.7)	0.334[b]	108 (59.7)	149 (56.0)	257 (57.5)	0.503[b]
Secondary-Superior	53 (31.2)	119 (36.0)	172 (34.3)		73 (40.3)	117 (44.0)	190 (42.5)	
**Smoking[2]**								
Non-smoker	120 (71.0)	207 (62.3)	327 (65.3)	0.120[b]	52 (28.4)	60 (22.3)	112 (24.8)	0.074[b]
Smoker	15 (8.9)	46 (13.9)	61 (12.2)		22 (12.0)	52 (19.3)	74 (16.4)	
Ex-smoker	34 (20.1)	79 (23.8)	113 (22.6)		109 (59.6)	157 (58.4)	266 (58.8)	
**Sedentary lifestyle[2]**	123 (72.4)	218 (65.5)	341 (67.8)	0.144[b]	114 (62.6)	174 (64.9)	288 (64.0)	0.692[b]
**Hypertension[2]**	76 (44.4)	157 (46.9)	233 (46.0)	0.673[b]	95 (51.4)	132 (49.1)	227 (50.0)	0.702[b]
**Diabetes Mellitus[2]**	21 (12.3)	46 (13.7)	67 (13.2)	0.751[b]	46 (24.9)	70 (26.0)	116 (25.6)	0.866[b]
**Dyslipidemia[2]**	88 (51.5)	172 (51.3)	260 (51.4)	1.000[b]	94 (50.8)	142 (52.8)	236 (52.0)	0.750[b]
**Atrial fibrillation[2]**	2 (1.2)	11 (3.3)	13 (2.6)	0.235[c]	13 (7.0)	22 (8.2)	35 (7.7)	0.785[b]
**Depression[2]**	35 (20.5)	57 (17.0)	92 (18.2)	0.406[b]	20 (10.8)	16 (5.9)	36 (7.9)	0.088[b]
**CNS treatment[1]**	53 (31.0)	99 (29.6)	152 (30.0)	0.816[b]	37 (20.0)	45 (16.7)	82 (18.1)	0.444[b]
**BMI[1]**	27.4 [24.9–29.8]	27.7 [24.8–30.9]	27.6 [24.8–30.5]	0.356[a]	28.7 [26.8–30.3]	28.6 [26.5–31.1]	28.7 [26.6–30.8]	0.427[a]
**SBP[1]**	130.0 [120.0–140.0]	130.0 [120.0–140.0]	130.0 [120.0–140.0]	0.591[a]	131.0 [120.0–141.0]	134.0 [124.0–142.0]	132.0 [121.0–142.0]	0.355[a]
**DBP[1]**	75.0 [70.0–80.0]	75.0 [70.0–80.0]	75.0 [70.0–80.0]	0.466[a]	76.0 [70.0–83.0]	78.00 [70.0–85.0]	77.0 [70.0–85.0]	0.481[a]
**Total cholesterol[1]**	205.0 [183.0–231.0]	208.0 [182.0–230.8]	208.0 [182.0–231.0]	0.729[a]	188.0 [161.8–211.0]	185.00 [159.0–213.0]	186.0 [160.0–212.0]	0.752[a]
**HDL–c[1]**	57.0 [49.0–68.0]	57.0 [49.0–66.0]	57.0 [49.0–67.0]	0.897[a]	49.0 [40.0–59.3]	46.00 [39.5–55.0]	48.0 [40.0–56.0]	0.071[a]
**REGICOR[2]**								
Low CVR	111 (76.6)	248 (82.1)	359 (80.3)	0.248[c]	68 (46.6)	79 (39.5)	147 (42.5)	0.238[b]
Moderate CVR	33 (22.8)	50 (16.6)	83 (18.6)		56 (38.4)	95 (47.5)	151 (43.6)	
High CVR	1 (0.7)	4 (1.3)	5 (1.1)		22 (15.1)	26 (13.0)	48 (13.9)	
**FRESCO[2]**								
Low CVR	60 (53.6)	124 (64.9)	184 (60.7)	0.008[b]	23 (21.5)	44 (30.6)	67 (26.7)	0.100[b]
Moderate CVR	38 (33.9)	60 (31.4)	98 (32.3)		46 (43.0)	65 (45.1)	111 (44.2)	
High CVR	14 (12.5)	7 (3.7)	21 (6.9)		38 (35.5)	35 (24.3)	73 (29.1)	

BMI: Body mass index; SBP: Systolic blood pressure (mmHg); DBP: Diastolic blood pressure (mmHg); HDL-c: High Density Lipoprotein cholesterol; CNS treatment: treatments that modulate the central nervous system; CVR: Cardiovascular risk. 1: median [Q1–Q3]; 2: n (%); a: Mann-Whitney U test; b: Chi-squared test; c: Fisher's test.
