# Supplementary material for: Cardiovascular risk and cognitive performance: A population-based cross-sectional study (NEDICES2-RISK)
Source: PLoS One. 2026 Mar 25;21(3):e0345086. doi: 10.1371/journal.pone.0345086 (PMC13016341; doi:10.1371/journal.pone.0345086)
Supplement: S4 Table — Comparison between participants with the worst score in the Delayed Recall test and the rest. (PDF) [file pone.0345086.s005.pdf]

S4 Table. Baseline characteristics of the sample and cardiovascular risk. Comparison between participants with the worst score in the Delayed Recall test and the rest.

	Women				Men			
	**≤P25 (n=183)**	**>P25 (n=322)**	**Overall (N=505)**	***p***	**≤P25 (n=182)**	**>P25 (n=273)**	**Overall (N=455)**	***p***
**Age[1]**	69.0 [64.0–72.0]	66.0 [61.3–71.0]	67.0 [62.0–71.0]	0.002[a]	68.5 [65.0–72.0]	65.0 [61.0–70.0]	67.0 [62.0–71.0]	<0.001[a]
**Education level[2]**								
No education-Primary	120 (66.7)	208 (65.0)	328 (65.6)	0.781[b]	107 (60.8)	150 (55.1)	257 (57.4)	0.279[b]
Secondary-Superior	60 (33.3)	112 (35.0)	172 (34.4)		69 (39.2)	122 (44.9)	191 (42.6)	
**Smoking[2]**								
Non-smoker	126 (69.6)	200 (62.7)	326 (65.2)	0.288[b]	51 (28.3)	62 (22.7)	113 (24.9)	0.106[b]
Smoker	20 (11.0)	41 (12.9)	61 (12.2)		22 (12.2)	52 (19.0)	74 (16.3)	
Ex-smoker	35 (19.3)	78 (24.5)	113 (22.6)		107 (59.4)	159 (58.2)	266 (58.7)	
**Sedentary lifestyle[2]**	132 (72.5)	209 (65.3)	341 (67.9)	0.117[b]	111 (62.0)	177 (65.1)	288 (63.9)	0.574[b]
**Hypertension[2]**	80 (43.7)	152 (47.2)	232 (45.9)	0.507[b]	95 (52.2)	132 (48.4)	227 (49.9)	0.479[b]
**Diabetes Mellitus[2]**	23 (12.6)	44 (13.7)	67 (13.3)	0.832[b]	42 (23.1)	74 (27.1)	116 (25.5)	0.392[b]
**Dyslipidemia[2]**	88 (48.1)	172 (53.4)	260 (51.5)	0.290[b]	88 (48.4)	148 (54.2)	236 (51.9)	0.258[b]
**Atrial fibrillation[2]**	4 (2.2)	9 (2.8)	13 (2.6)	0.777[c]	12 (6.6)	23 (8.4)	35 (7.7)	0.590[b]
**Depression[2]**	40 (21.9)	52 (16.1)	92 (18.2)	0.139[b]	19 (10.4)	17 (6.2)	36 (7.9)	0.146[b]
**CNS treatment[1]**	67 (36.6)	85 (26.4)	152 (30.1)	0.021[b]	32 (17.6)	50 (18.3)	82 (18.0)	0.940[b]
**BMI[1]**	27.6 [25.1–30.0]	27.6 [24.6–30.9]	27.6 [24.8–30.5]	0.849[a]	29.1 [27.2–30.3]	28.4 [26.2–30.9]	28.7 [26.6–30.8]	0.394[a]
**SBP[1]**	130.0 [120.0–140.0]	130.0 [120.0–140.0]	130.0 [120.0–140.0]	0.693[a]	132.0 [120.3–140.0]	132.0 [121.0–143.0]	132.0 [120.5–142.0]	0.361[a]
**DBP[1]**	75.0 [70.0–80.0]	76.0 [70.0–80.0]	75.0 [70.0–80.0]	0.100[a]	77.0 [70.0–85.0]	77.0 [70.0–85.0]	77.0 [70.0–85.0]	0.584[a]
**Total cholesterol[1]**	206.5 [183.3–232.8]	208.0 [182.0–228.8]	208.0 [182.0–231.0]	0.349[a]	185.0 [162.0–211.0]	186.0 [157.8–213.0]	186.0 [160.0–212.0]	0.898[a]
**HDL–c[1]**	57.0 [49.0–69.0]	57.0 [48.3–66.8]	57.0 [49.0–67.0]	0.288[a]	49.0 [40.8–60.0]	47.0 [39.8–54.0]	48.0 [40.0–56.3]	0.104[a]
**REGICOR[2]**								
Low CVR	123 (82.0)	235 (79.4)	358 (80.3)	0.771[c]	64 (46.7)	84 (40.0)	148 (42.7)	0.456[b]
Moderate CVR	26 (17.3)	57 (19.3)	83 (18.6)		56 (40.9)	95 (45.2)	151 (43.5)	
High CVR	1 (0.7)	4 (1.4)	5 (1.1)		17 (12.4)	31 (14.8)	48 (13.8)	
**FRESCO[2]**								
Low CVR	67 (58.3)	116 (62.0)	183 (60.6)	0.065[b]	29 (27.1)	39 (26.9)	68 (27.0)	0.099[b]
Moderate CVR	35 (30.4)	63 (33.7)	98 (32.5)		40 (37.4)	71 (49.0)	111 (44.0)	
High CVR	13 (11.3)	8 (4.3)	21 (7.0)		38 (35.5)	35 (24.1)	73 (29.0)	

BMI: Body mass index; SBP: Systolic blood pressure (mmHg); DBP: Diastolic blood pressure (mmHg); HDL-c: High Density Lipoprotein cholesterol; CNS treatment: treatments that modulate the central nervous system; CVR: Cardiovascular risk. 1: median [Q1–Q3]; 2: n (%); a: Mann-Whitney U test; b: Chi-squared test; c: Fisher's test.
