# Supplementary material for: Cardiovascular risk and cognitive performance: A population-based cross-sectional study (NEDICES2-RISK)
Source: PLoS One. 2026 Mar 25;21(3):e0345086. doi: 10.1371/journal.pone.0345086 (PMC13016341; doi:10.1371/journal.pone.0345086)
Supplement: S5 Table — Comparison between participants with the worst score in the Word Accentuation test and the rest. (PDF) [file pone.0345086.s006.pdf]

S5 Table. Baseline characteristics of the sample and cardiovascular risk. Comparison between participants with the worst score in the Word Accentuation test and the rest.

	Women				Men			
	**≤P25 (n=132)**	**>P25 (n=366)**	**Overall (N=498)**	***p***	**≤P25 (n=128)**	**>P25 (n=318)**	**Overall (N=446)**	***p***
**Age**[1]	70.0 [65.0–72.0]	66.0 [61.0–71.0]	67.0 [62.0–71.0]	<0.001[a]	70.0 [64.0–73.0]	65.0 [61.0–70.0]	67.0 [62.0–71.0]	<0.001[a]
**Education level**[2]								
No education-Primary	123 (94.6)	200 (54.9)	323 (65.4)	<0.001[b]	116 (92.1)	134 (42.7)	250 (56.8)	<0.001[b]
Secondary-Superior	7 (5.4)	164 (45.1)	171 (34.6)		10 (7.9)	180 (57.3)	190 (43.2)	
**Smoking**[2]								
Non-smoker	106 (80.3)	216 (59.7)	322 (65.2)	<0.001[b]	42 (33.1)	71 (22.3)	113 (25.4)	0.063[b]
Smoker	10 (7.6)	51 (14.1)	61 (12.3)		19 (15.0)	55 (17.3)	74 (16.6)	
Ex-smoker	16 (12.1)	95 (26.2)	111 (22.5)		66 (52.0)	192 (60.4)	258 (58.0)	
**Sedentary lifestyle**[2]	106 (80.3)	230 (63.2)	336 (67.7)	<0.001[b]	83 (65.4)	198 (62.9)	281 (63.6)	0.701[b]
**Hypertension**[2]	78 (59.1)	149 (40.7)	227 (45.6)	<0.001[b]	67 (52.3)	155 (48.7)	222 (49.8)	0.560[b]
**Diabetes Mellitus**[2]	26 (19.7)	41 (11.2)	67 (13.5)	0.021[b]	35 (27.3)	79 (24.8)	114 (25.6)	0.669[b]
**Dyslipidemia**[2]	65 (49.2)	192 (52.5)	257 (51.6)	0.594[b]	57 (44.5)	174 (54.7)	231 (51.8)	0.065[b]
**Atrial fibrillation**[2]	4 (3.0)	9 (2.5)	13 (2.6)	0.752[c]	9 (7.0)	26 (8.2)	35 (7.8)	0.832[b]
**Depression**[2]	24 (18.2)	68 (18.6)	92 (18.5)	1.000[b]	11 (8.6)	25 (7.9)	36 (8.1)	0.948[b]
**CNS treatment**[1]	41 (31.1)	110 (30.1)	151 (30.3)	0.916[b]	20 (15.6)	61 (19.2)	81 (18.2)	0.456[b]
**BMI**[1]	28.9 [26.3–32.8]	26.7 [24.4–30.0]	27.5 [24.8–30.5]	<0.001[a]	29.1 [27.3–31.1]	28.4 [26.4–30.5]	28.7 [26.5–30.8]	0.093[a]
**SBP**[1]	130.0 [120.0–140.0]	130.0 [120.0–140.0]	130.0 [120.0–140.0]	0.123[a]	135.0 [121.0–145.0]	131.5 [120.0–140.0]	132.0 [120.0–141.8]	0.180[a]
**DBP**[1]	75.0 [70.0–80.0]	75.0 [70.0–80.0]	75.0 [70.0–80.0]	0.768[a]	76.5 [70.0–83.0]	77.0 [70.0–85.0]	77.0 [70.0–85.0]	0.646[a]
**Total cholesterol**[1]	198.5 [172.8–224.5]	210.0 [185.0–231.0]	208.0 [182.0–230.0]	0.010[a]	189.0 [164.5–213.0]	184.0 [157.0–211.3]	186.0 [160.5–212.5]	0.408[a]
**HDL–c**[1]	56.0 [47.0–63.0]	58.0 [50.0–69.0]	57.0 [49.0–67.0]	0.020[a]	48.0 [38.0–54.0]	48.0 [40.0–58.0]	48.0 [40.0–56.0]	0.228[a]
**REGICOR**[2]								
Low CVR	83 (74.8)	269 (81.8)	352 (80.0)	0.212[c]	28 (28.9)	116 (47.5)	144 (42.2)	0.001[b]
Moderate CVR	26 (23.4)	57 (17.3)	83 (18.9)		47 (48.5)	102 (41.8)	149 (43.7)	
High CVR	2 (1.8)	3 (0.9)	5 (1.1)		22 (22.7)	26 (10.7)	48 (14.1)	
**FRESCO**[2]								
Low CVR	40 (50.0)	139 (64.1)	179 (60.3)	0.010[b]	11 (14.3)	55 (32.2)	66 (26.6)	0.002[b]
Moderate CVR	29 (36.3)	68 (31.3)	97 (32.7)		34 (44.2)	76 (44.4)	110 (44.4)	
High CVR	11 (13.8)	10 (4.6)	21 (7.1)		32 (41.6)	40 (23.4)	72 (29.0)	

BMI: Body mass index; SBP: Systolic blood pressure (mmHg); DBP: Diastolic blood pressure (mmHg); HDL-c: High Density Lipoprotein cholesterol; CNS treatment: treatments that modulate the central nervous system; CVR: Cardiovascular risk. 1: median [Q1–Q3]; 2: n (%); a: Mann-Whitney U test; b: Chi-squared test; c: Fisher's test.
