# Supplementary material for: Cardiovascular risk and cognitive performance: A population-based cross-sectional study (NEDICES2-RISK)
Source: PLoS One. 2026 Mar 25;21(3):e0345086. doi: 10.1371/journal.pone.0345086 (PMC13016341; doi:10.1371/journal.pone.0345086)
Supplement: S6 Table — Comparison between participants with the worst score in the Verbal Fluency test and the rest. (PDF) [file pone.0345086.s007.pdf]

S6 Table. Baseline characteristics of the sample and cardiovascular risk. Comparison between participants with the worst score in the Verbal Fluency test and the rest.

	**Women**				**Men**			
	**≤P25 (n=166)**	**>P25 (n=337)**	**Overall (N=503)**	***p***	**≤P25 (n=114)**	**>P25 (n=341)**	**Overall (N=455)**	***p***
**Age[1]**	70.0 [65.3–72.0]	66.0 [61.0–71.0]	67.0 [62.0–71.0]	<0.001[a]	70.0 [66.3–73.0]	65.0 [61.0–71.0]	67.0 [62.0–71.0]	<0.001[a]
**Education level[2]**								
No education-Primary	127 (77.9)	199 (59.4)	326 (65.5)	<0.001[b]	85 (75.2)	172 (51.3)	257 (57.4)	<0.001[b]
Secondary-Superior	36 (22.1)	136 (40.6)	172 (34.5)		28 (24.8)	163 (48.7)	191 (42.6)	
**Smoking[2]**								
Non-smoker	126 (77.3)	198 (59.1)	324 (65.1)	<0.001[b]	26 (23.2)	87 (25.5)	113 (24.9)	0.630[b]
Smoker	9 (5.5)	52 (15.5)	61 (12.2)		16 (14.3)	58 (17.0)	74 (16.3)	
Ex-smoker	28 (17.2)	85 (25.4)	113 (22.7)		70 (62.5)	196 (57.5)	266 (58.7)	
**Sedentary lifestyle[2]**	126 (76.4)	213 (63.6)	339 (67.8)	0.006[b]	78 (69.6)	210 (61.9)	288 (63.9)	0.175[b]
**Hypertension[2]**	96 (57.8)	136 (40.4)	232 (46.1)	<0.001[b]	63 (55.3)	164 (48.1)	227 (49.9)	0.224[b]
**Diabetes Mellitus[2]**	31 (18.7)	37 (11.0)	68 (13.5)	0.025[b]	40 (35.1)	76 (22.3)	116 (25.5)	0.010[b]
**Dyslipidemia[2]**	79 (47.6)	180 (53.4)	259 (51.5)	0.257[b]	58 (50.9)	178 (52.2)	236 (51.9)	0.892[b]
**Atrial fibrillation[2]**	7 (4.2)	6 (1.8)	13 (2.6)	0.187[b]	7 (6.1)	28 (8.2)	35 (7.7)	0.606[b]
**Depression[2]**	30 (18.1)	61 (18.1)	91 (18.1)	1.000[b]	10 (8.8)	26 (7.6)	36 (7.9)	0.847[b]
**CNS treatment[1]**	55 (33.1)	96 (28.5)	151 (30.0)	0.334[b]	21 (18.4)	61 (17.9)	82 (18.0)	1.000[b]
**BMI[1]**	27.9 [25.7–31.1]	26.9 [24.5–30.2]	27.5 [24.8–30.4]	0.016[a]	28.8 [27.3–30.5]	28.6 [26.4–30.8]	28.7 [26.6–30.8]	0.412[a]
**SBP[1]**	130.0 [120.0–140.0]	130.0 [120.0–140.0]	130.0 [120.0–140.0]	0.172[a]	135.0 [124.3–143.8]	131.0 [120.0–140.0]	132.0 [120.5–142.0]	0.165[a]
**DBP[1]**	75.0 [70.0–80.0]	75.0 [70.0–80.0]	75.0 [70.0–80.0]	0.223[a]	75.5 [68.0–85.0]	78.0 [70.0–85.0]	77.0 [70.0–85.0]	0.228[a]
**Total cholesterol[1]**	200.5 [175.0–228.0]	210.0 [185.8–231.0]	208.0 [182.3–230.8]	0.018[a]	186.5 [155.5–208.8]	186.0 [161.0–213.0]	186.0 [160.0–212.0]	0.489[a]
**HDL–c[1]**	59.0 [48.0–69.0]	56.0 [49.0–66.0]	57.0 [49.0–67.0]	0.162[a]	48.5 [40.0–58.8]	48.0 [40.0–55.0]	48.0 [40.0–56.3]	0.328[a]
**REGICOR[2]**								
Low CVR	108 (79.4)	248 (80.3)	356 (80.0)	0.392[c]	27 (33.3)	121 (45.5)	148 (42.7)	0.143[b]
Moderate CVR	25 (18.4)	59 (19.1)	84 (18.9)		40 (49.4)	111 (41.7)	151 (43.5)	
High CVR	3 (2.2)	2 (0.6)	5 (1.1)		14 (17.3)	34 (12.8)	48 (13.8)	
**FRESCO[2]**								
Low CVR	63 (60.0)	120 (61.2)	183 (60.8)	0.434[b]	12 (21.8)	56 (28.4)	68 (27.0)	0.483[b]
Moderate CVR	32 (30.5)	65 (33.2)	97 (32.2)		24 (43.6)	87 (44.2)	111 (44.0)	
High CVR	10 (9.5)	11 (5.6)	21 (7.0)		19 (34.5)	54 (27.4)	73 (29.0)	

BMI: Body mass index; SBP: Systolic blood pressure (mmHg); DBP: Diastolic blood pressure (mmHg); HDL-c: High Density Lipoprotein cholesterol; CNS treatment: treatments that modulate the central nervous system; CVR: Cardiovascular risk. 1: median [Q1–Q3]; 2: n (%); a: Mann-Whitney U test; b: Chi-squared test; c: Fisher's test.
