# Supplementary material for: Cardiovascular risk and cognitive performance: A population-based cross-sectional study (NEDICES2-RISK)
Source: PLoS One. 2026 Mar 25;21(3):e0345086. doi: 10.1371/journal.pone.0345086 (PMC13016341; doi:10.1371/journal.pone.0345086)
Supplement: S7 Table — Comparison between participants with the worst score in the Clock drawing test and the rest. (PDF) [file pone.0345086.s008.pdf]

S7 Table. Baseline characteristics of the sample and cardiovascular risk. Comparison between participants with the worst score in the Clock Drawing test and the rest.

	**Women**				**Men**			
	**≤P25 (n=128)**	**>P25 (n=267)**	**Overall (N=395)**	***p***	**≤P25 (n=119)**	**>P25 (n=246)**	**Overall (N=365)**	***p***
**Age[1]**	68.5 [65.0–72.0]	65.0 [61.0–70.0]	66.0 [61.0–71.0]	<0.001[a]	69.0 [63.5–73.0]	65.0 [61.0–70.0]	67.0 [62.0–71.0]	<0.001[a]
**Education level[2]**								
No education-Primary	109 (86.5)	160 (60.4)	269 (68.8)	<0.001[b]	98 (83.1)	116 (48.1)	214 (59.6)	<0.001[b]
Secondary-Superior	17 (13.5)	105 (39.6)	122 (31.2)		20 (16.9)	125 (51.9)	145 (40.4)	
**Smoking[2]**								
Non-smoker	101 (80.2)	160 (60.4)	261 (66.8)	<0.001[b]	38 (32.2)	54 (22.0)	92 (25.3)	0.104[b]
Smoker	7 (5.6)	46 (17.4)	53 (13.6)		17 (14.4)	44 (17.9)	61 (16.8)	
Ex-smoker	18 (14.3)	59 (22.3)	77 (19.7)		63 (53.4)	148 (60.2)	211 (58.0)	
**Sedentary lifestyle[2]**	96 (75.6)	165 (62.3)	261 (66.6)	0.012[b]	76 (64.4)	146 (59.6)	222 (61.2)	0.443[b]
**Hypertension[2]**	77 (60.2)	102 (38.2)	179 (45.3)	<0.001[b]	61 (51.3)	120 (48.8)	181 (49.6)	0.739[b]
**Diabetes Mellitus[2]**	19 (14.8)	32 (12.0)	51 (12.9)	0.527[b]	35 (29.4)	61 (24.8)	96 (26.3)	0.417[b]
**Dyslipidemia[2]**	69 (53.9)	131 (49.1)	200 (50.6)	0.428[b]	51 (42.9)	132 (53.7)	183 (50.1)	0.068[b]
**Atrial fibrillation[2]**	4 (3.1)	7 (2.6)	11 (2.8)	0.753[c]	9 (7.6)	21 (8.5)	30 (8.2)	0.909[b]
**Depression[2]**	26 (20.3)	48 (18.0)	74 (18.7)	0.675[b]	14 (11.8)	13 (5.3)	27 (7.4)	0.045[b]
**CNS treatment[1]**	46 (35.9)	69 (25.8)	115 (29.1)	0.051[b]	23 (19.3)	42 (17.1)	65 (17.8)	0.703[b]
**BMI[1]**	27.9 [25.1–31.2]	27.0 [24.5–30.4]	27.4 [24.7–30.5]	0.098[a]	29.4 [26.4–31.6]	28.6 [26.7–30.5]	28.7 [26.5–30.9]	0.308[a]
**SBP[1]**	130.0 [120.0–142.0]	130.0 [120.0–140.0]	130.0 [120.0–140.0]	0.155[a]	134.0 [120.0–145.0]	132.0 [121.3–140.0]	132.0 [121.0–142.0]	0.643[a]
**DBP[1]**	77.0 [70.0–81.3]	75.0 [70.0–80.0]	75.0 [70.0–80.0]	0.300[a]	78.0 [70.0–85.0]	78.0 [70.0–84.8]	78.0 [70.0–85.0]	0.742[a]
**Total cholesterol[1]**	207.5 [179.8–228.0]	209.0 [184.0–235.0]	209.0 [183.0–231.0]	0.188[a]	189.0 [167.0–212.8]	187.0 [160.8–215.0]	188.0 [163.0–215.0]	0.809[a]
**HDL–c[1]**	54.0 [47.0–64.0]	57.5 [49.0–69.0]	56.0 [48.0–67.0]	0.018[a]	47.0 [38.0–53.0]	47.0 [39.8–55.0]	47.0 [39.0–55.0]	0.788[a]
**REGICOR[2]**								
Low CVR	80 (75.5)	198 (80.2)	278 (78.8)	0.226[c]	32 (36.4)	81 (41.8)	113 (40.1)	0.510[b]
Moderate CVR	26 (24.5)	45 (18.2)	71 (20.1)		39 (44.3)	85 (43.8)	124 (44.0)	
High CVR	0 (0)	4 (1.6)	4 (1.1)		17 (19.3)	28 (14.4)	45 (16.0)	
**FRESCO[2]**								
Low CVR	35 (46.1)	114 (68.7)	149 (61.6)	0.002[b]	16 (22.9)	41 (28.9)	57 (26.9)	0.164[b]
Moderate CVR	31 (40.8)	43 (25.9)	74 (30.6)		28 (40.0)	66 (46.5)	94 (44.3)	
High CVR	10 (13.2)	9 (5.4)	19 (7.9)		26 (37.1)	35 (24.6)	61 (28.8)	

BMI: Body mass index; SBP: Systolic blood pressure (mmHg); DBP: Diastolic blood pressure (mmHg); HDL-c: High Density Lipoprotein cholesterol; CNS treatment: treatments that modulate the central nervous system; CVR: Cardiovascular risk. 1: median [Q1–Q3]; 2: n (%); a: Mann-Whitney U test; b: Chi-squared test; c: Fisher's test.
