# Supplementary material for: Cardiovascular risk and cognitive performance: A population-based cross-sectional study (NEDICES2-RISK)
Source: PLoS One. 2026 Mar 25;21(3):e0345086. doi: 10.1371/journal.pone.0345086 (PMC13016341; doi:10.1371/journal.pone.0345086)
Supplement: S8 Table — Comparison between participants with the worst score in the TMTA-1 and the rest. (PDF) [file pone.0345086.s009.pdf]

S8 Table. Baseline characteristics of the sample and cardiovascular risk. Comparison between participants with the worst score in the TMTA-1 and the rest.

	**Women**				**Men**			
	**≥P75 (n=140)**	**P75 (n=359)**	**Overall (N=499)**	***p***	**≥P75 (n=105)**	**<P75 (n=341)**	**Overall (N=446)**	***p***
**Age[1]**	71.0 [66.0–73.0]	66.0 [61.0–70.0]	67.0 [62.0–71.0]	<0.001[a]	71.0 [67.0–74.0]	65.0 [61.0–70.0]	67.0 [62.0–71.0]	<0.001[a]
**Education level[2]**								
No education-Primary	119 (86.2)	208 (58.4)	327 (66.2)	<0.001[b]	91 (87.5)	160 (47.6)	251 (57.0)	<0.001[b]
Secondary-Superior	19 (13.8)	148 (41.6)	167 (33.8)		13 (12.5)	176 (52.4)	189 (43.0)	
**Smoking[2]**								
Non-smoker	114 (82.0)	209 (58.9)	323 (65.4)	<0.001[b]	38 (36.2)	75 (22.1)	113 (25.5)	0.014[b]
Smoker	12 (8.6)	49 (13.8)	61 (12.3)		16 (15.2)	57 (16.8)	73 (16.4)	
Ex-smoker	13 (9.4)	97 (27.3)	110 (22.3)		51 (48.6)	207 (61.1)	258 (58.1)	
**Sedentary lifestyle[2]**	112 (80.0)	224 (62.9)	336 (67.7)	<0.001[b]	74 (71.2)	205 (60.7)	279 (63.1)	0.068[b]
**Hypertension[2]**	74 (52.9)	157 (43.7)	231 (46.3)	0.083[b]	55 (52.4)	165 (48.4)	220 (49.3)	0.546[b]
**Diabetes Mellitus[2]**	27 (19.3)	39 (10.9)	66 (13.2)	0.019[b]	34 (32.4)	80 (23.5)	114 (25.6)	0.088[b]
**Dyslipidemia[2]**	71 (50.7)	187 (52.1)	258 (51.7)	0.860[b]	49 (46.7)	181 (53.1)	230 (51.6)	0.299[b]
**Atrial fibrillation[2]**	4 (2.9)	9 (2.5)	13 (2.6)	0.763[c]	12 (11.4)	22 (6.5)	34 (7.6)	0.142[b]
**Depression[2]**	31 (22.1)	59 (16.4)	90 (18.0)	0.174[b]	13 (12.4)	21 (6.2)	34 (7.6)	0.059[b]
**CNS treatment[1]**	44 (31.4)	105 (29.2)	149 (29.9)	0.712[b]	24 (22.9)	55 (16.1)	79 (17.7)	0.152[b]
**BMI[1]**	28.2 [25.7–30.9]	27.3 [24.6–30.4]	27.6 [24.8–30.6]	0.061[a]	29.3 [27.3–31.1]	28.5 [26.4–30.5]	28.7 [26.6–30.8]	0.111[a]
**SBP[1]**	132.0 [120.0–142.3]	130.0 [120.0–140.0]	130.0 [120.0–140.0]	0.009[a]	133.0 [120.0–145.0]	132.0 [120.0–140.0]	132.0 [120.0–141.0]	0.490[a]
**DBP[1]**	75.0 [70.0–80.0]	75.0 [70.0–80.0]	75.0 [70.0–80.0]	0.552[a]	75.0 [70.0–80.0]	77.0 [70.0–85.0]	77.0 [70.0–85.0]	0.139[a]
**Total cholesterol[1]**	207.0 [182.5–234.3]	208.0 [182.3–229.0]	207.5 [182.3–230.8]	0.590[a]	185.5 [159.5–204.3]	186.0 [160.0–214.0]	186.0 [160.0–212.0]	0.549[a]
**HDL–c[1]**	56.0 [47.0–64.0]	58.0 [50.0–68.0]	57.0 [49.0–67.0]	0.015[a]	47.0 [37.8–55.3]	48.0 [41.0–57.0]	48.0 [40.0–56.5]	0.215[a]
**REGICOR[2]**								
Low CVR	84 (79.2)	269 (80.5)	353 (80.2)	0.185[c]	17 (23.3)	130 (48.3)	147 (43.0)	<0.001[b]
Moderate CVR	19 (17.9)	63 (18.9)	82 (18.6)		39 (53.4)	108 (40.1)	147 (43.0)	
High CVR	3 (2.8)	2 (0.6)	5 (1.1)		17 (23.3)	31 (11.5)	48 (14.0)	
**FRESCO[2]**								
Low CVR	29 (37.2)	151 (68.6)	180 (60.4)	<0.001[b]	8 (12.9)	60 (32.3)	68 (27.4)	<0.001[b]
Moderate CVR	37 (47.4)	60 (27.3)	97 (32.6)		26 (41.9)	84 (45.2)	110 (44.4)	
High CVR	12 (15.4)	9 (4.1)	21 (7.0)		28 (45.2)	42 (22.6)	70 (28.2)	

TMTA: Trail making test series A (seconds); BMI: Body mass index; SBP: Systolic blood pressure (mmHg); DBP: Diastolic blood pressure (mmHg); HDL-c: High Density Lipoprotein cholesterol; CNS treatment: treatments that modulate the central nervous system; CVR: Cardiovascular risk. 1: median [Q1–Q3]; 2: n (%); a: Mann-Whitney U test; b: Chi-squared test; c: Fisher's test.
