# Supplementary material for: Cardiovascular risk and cognitive performance: A population-based cross-sectional study (NEDICES2-RISK)
Source: PLoS One. 2026 Mar 25;21(3):e0345086. doi: 10.1371/journal.pone.0345086 (PMC13016341; doi:10.1371/journal.pone.0345086)
Supplement: S9 Table — Comparison between participants with the worst score in the TMTA-2 and the rest. (PDF) [file pone.0345086.s010.pdf]

S9 Table. Baseline characteristics of the sample and cardiovascular risk. Comparison between participants with the worst score in the TMTA-2 and the rest.

	Women				Men			
	**≥P75 (n=133)**	**<P75 (n=365)**	**Overall (N=498)**	***p***	**≥P75 (n=103)**	**<P75 (n=342)**	**Overall (N=445)**	***p***
**Age[1]**	70.0 [64.0–73.0]	67.0 [62.0–71.0]	67.0 [62.0–71.0]	<0.001[a]	70.0 [66.0–73.0]	65.0 [61.0–70.0]	67.0 [62.0–71.0]	<0.001[a]
**Education level[2]**								
No education-Primary	118 (90.8)	209 (57.6)	327 (66.3)	<0.001[b]	87 (86.1)	163 (48.2)	250 (56.9)	<0.001[b]
Secondary-Superior	12 (9.2)	154 (42.4)	166 (33.7)		14 (13.9)	175 (51.8)	189 (43.1)	
**Smoking[2]**								
Non-smoker	105 (80.2)	218 (60.2)	323 (65.5)	<0.001[b]	40 (39.2)	73 (21.4)	113 (25.5)	0.001[b]
Smoker	13 (9.9)	48 (13.3)	61 (12.4)		12 (11.8)	61 (17.9)	73 (16.5)	
Ex-smoker	13 (9.9)	96 (26.5)	109 (22.1)		50 (49.0)	207 (60.7)	257 (58.0)	
**Sedentary lifestyle[2]**	110 (83.3)	225 (62.0)	335 (67.7)	<0.001[b]	66 (64.1)	212 (62.7)	278 (63.0)	0.894[b]
**Hypertension[2]**	78 (58.6)	152 (41.6)	230 (46.2)	0.001[b]	47 (45.6)	172 (50.3)	219 (49.2)	0.473[b]
**Diabetes Mellitus[2]**	28 (21.1)	38 (10.4)	66 (13.3)	0.003[b]	34 (33.0)	79 (23.1)	113 (25.4)	0.058[b]
**Dyslipidemia[2]**	68 (51.1)	190 (52.1)	258 (51.8)	0.935[b]	43 (41.7)	187 (54.7)	230 (51.7)	0.029[b]
**Atrial fibrillation[2]**	3 (2.3)	10 (2.7)	13 (2.6)	1.000[c]	11 (10.7)	23 (6.7)	34 (7.6)	0.266[b]
**Depression[2]**	31 (23.3)	59 (16.2)	90 (18.1)	0.089[b]	11 (10.7)	23 (6.7)	34 (7.6)	0.266[b]
**CNS treatment[1]**	46 (34.6)	103 (28.2)	149 (29.9)	0.207[b]	20 (19.4)	59 (17.3)	79 (17.8)	0.721[b]
**BMI[1]**	28.2 [25.6–31.5]	27.3 [24.7–30.2]	27.6 [24.8–30.6]	0.014[a]	29.1 [27.2–31.4]	28.6 [26.4–30.5]	28.7 [26.6–30.8]	0.087[a]
**SBP[1]**	132.0 [121.0–145.0]	130.0 [120.0–140.0]	130.0 [120.0–140.0]	0.004[a]	135.0 [120.5–143.5]	131.5 [120.0–140.0]	132.0 [120.0–141.0]	0.497[a]
**DBP[1]**	75.0 [70.0–81.0]	75.0 [70.0–80.0]	75.0 [70.0–80.0]	0.456[a]	75.0 [70.0–80.0]	77.0 [70.0–85.0]	77.0 [70.0–85.0]	0.066[a]
**Total cholesterol[1]**	202.0 [177.0–228.0]	209.0 [184.0–231.0]	208.0 [183.0–231.0]	0.160[a]	183.5 [160.0–209.3]	187.5 [159.5–212.3]	186.0 [160.0–212.0]	0.634[a]
**HDL–c[1]**	55.5 [46.8–64.0]	58.0 [50.0–68.0]	57.0 [49.0–67.0]	0.013[a]	45.5 [38.0–54.0]	48.0 [40.8–57.3]	48.0 [40.0–56.0]	0.098[a]
**REGICOR[2]**								
Low CVR	81 (75.0)	272 (81.9)	353 (80.2)	0.076[c]	22 (28.6)	125 (47.2)	147 (43.0)	0.006[b]
Moderate CVR	24 (22.2)	58 (17.5)	82 (18.6)		38 (49.4)	109 (41.1)	147 (43.0)	
High CVR	3 (2.8)	2 (0.6)	5 (1.1)		17 (22.1)	31 (11.7)	48 (14.0)	
**FRESCO[2]**								
Low CVR	30 (42.9)	149 (65.6)	179 (60.3)	<0.001[b]	12 (18.5)	56 (30.8)	68 (27.5)	0.046[b]
Moderate CVR	30 (42.9)	67 (29.5)	97 (32.7)		28 (43.1)	82 (45.1)	110 (44.5)	
High CVR	10 (14.3)	11 (4.8)	21 (7.1)		25 (38.5)	44 (24.2)	69 (27.9)	

TMTA: Trail making test series A (seconds); BMI: Body mass index; SBP: Systolic blood pressure (mmHg); DBP: Diastolic blood pressure (mmHg); CNS treatment: treatments that modulate the central nervous system; HDL-c: High Density Lipoprotein cholesterol; CVR: Cardiovascular risk. 1: median [Q1–Q3]; 2: n (%); a: Mann-Whitney U test; b: Chi-squared test; c: Fisher's test.
