# Supplementary material for: Cardiovascular risk and cognitive performance: A population-based cross-sectional study (NEDICES2-RISK)
Source: PLoS One. 2026 Mar 25;21(3):e0345086. doi: 10.1371/journal.pone.0345086 (PMC13016341; doi:10.1371/journal.pone.0345086)
Supplement: S10 Table — Comparison between participants with the worst score in the TMTA-1 errors test and the rest. (PDF) [file pone.0345086.s011.pdf]

S10 Table. Baseline characteristics of the sample and cardiovascular risk. Comparison between participants with the worst score in the TMTA-Errors 1 and the rest.

	**Women**				**Men**			
	**≥P75 (n=147)**	**<P75 (n=355)**	**Overall (N=502)**	***p***	**≥P75 (n=104)**	**<P75 (n=342)**	**Overall (N=446)**	***p***
**Age[1]**	70.0 [66.0–72.5]	66.0 [61.0–71.0]	67.0 [62.0–71.0]	<0.001[a]	69.5 [65.0–73.0]	66.0 [61.0–71.0]	67.0 [62.0–71.0]	<0.001[a]
**Education level[2]**								
No education-Primary	105 (71.9)	223 (63.5)	328 (66.0)	0.090[b]	65 (63.1)	187 (55.5)	252 (57.3)	0.210[b]
Secondary-Superior	41 (28.1)	128 (36.5)	169 (34.0)		38 (36.9)	150 (44.5)	188 (42.7)	
**Smoking[2]**								
Non-smoker	98 (67.1)	227 (64.7)	325 (65.4)	0.679[b]	32 (31.1)	81 (23.8)	113 (25.5)	0.117[b]
Smoker	15 (10.3)	46 (13.1)	61 (12.3)		20 (19.4)	52 (15.2)	72 (16.2)	
Ex-smoker	33 (22.6)	78 (22.2)	111 (22.3)		51 (49.5)	208 (61.0)	259 (58.3)	
**Sedentary lifestyle[2]**	117 (79.6)	220 (62.5)	337 (67.5)	<0.001[b]	76 (74.5)	204 (60.0)	280 (63.3)	0.011[b]
**Hypertension[2]**	80 (54.4)	151 (42.5)	231 (46.0)	0.020[b]	54 (51.9)	166 (48.5)	220 (49.3)	0.622[b]
**Diabetes Mellitus[2]**	24 (16.3)	43 (12.1)	67 (13.3)	0.263[b]	32 (30.8)	82 (24.0)	114 (25.6)	0.207[b]
**Dyslipidemia[2]**	77 (52.4)	182 (51.3)	259 (51.6)	0.897[b]	56 (53.8)	175 (51.2)	231 (51.8)	0.714[b]
**Atrial fibrillation[2]**	6 (4.1)	7 (2.0)	13 (2.6)	0.296[b]	7 (6.7)	27 (7.9)	34 (7.6)	0.857[b]
**Depression[2]**	23 (15.6)	69 (19.4)	92 (18.3)	0.383[b]	11 (10.6)	23 (6.7)	34 (7.6)	0.278[b]
**CNS treatment[1]**	55 (37.4)	96 (27.0)	151 (30.1)	0.028[a]	27 (26.0)	52 (15.2)	79 (17.7)	0.018[b]
**BMI[1]**	28.0 [24.0–30.8]	27.4 [25.0–30.4]	27.6 [24.8–30.6]	0.904[a]	29.2 [27.3–30.5]	28.6 [26.4–30.8]	28.7 [26.6–30.8]	0.320[a]
**SBP[1]**	130.0 [120.0–140.0]	130.0 [120.0–140.0]	130.0 [120.0–140.0]	0.189[a]	132.0 [120.0–140.3]	132.0 [120.0–141.0]	132.0 [120.0–141.0]	0.808[a]
**DBP[1]**	75.0 [70.0–80.0]	75.0 [70.0–80.0]	75.0 [70.0–80.0]	0.327[a]	75.0 [70.0–83.5]	78.0 [70.0–85.0]	77.0 [70.0–85.0]	0.409[a]
**Total cholesterol[1]**	202.0 [176.5–226.0]	209.0 [184.0–232.0]	208.0 [182.0–231.0]	0.129[a]	177.5 [149.3–204.0]	188.0 [162.0–214.0]	186.0 [160.0–212.0]	0.041[a]
**HDL–c[1]**	57.0 [48.0–65.8]	57.0 [49.0–68.0]	57.0 [49.0–67.0]	0.482[a]	47.0 [39.0–56.0]	48.0 [40.0–57.0]	48.0 [40.0–57.0]	0.971[a]
**REGICOR[2]**								
Low CVR	99 (81.8)	256 (79.5)	355 (80.1)	0.879[c]	34 (47.2)	114 (42.2)	148 (43.3)	0.107[b]
Moderate CVR	21 (17.4)	62 (19.3)	83 (18.7)		24 (33.3)	123 (45.6)	147 (43.0)	
High CVR	1 (0.8)	4 (1.2)	5 (1.1)		14 (19.4)	33 (12.2)	47 (13.7)	
**FRESCO[2]**								
Low CVR	43 (52.4)	139 (63.8)	182 (60.7)	0.171[b]	14 (25.0)	54 (28.1)	68 (27.4)	0.323[b]
Moderate CVR	31 (37.8)	66 (30.3)	97 (32.3)		22 (39.3)	89 (46.4)	111 (44.8)	
High CVR	8 (9.8)	13 (6.0)	21 (7.0)		20 (35.7)	49 (25.5)	69 (27.8)	

TMTA: Trail making test series A (seconds); BMI: Body mass index; SBP: Systolic blood pressure (mmHg); DBP: Diastolic blood pressure (mmHg); HDL-c: High Density Lipoprotein cholesterol; CNS treatment: treatments that modulate the central nervous system; CVR: Cardiovascular risk. 1: median [Q1–Q3]; 2: n (%); a: Mann-Whitney U test; b: Chi-squared test; c: Fisher's test.
