# Supplementary material for: Cardiovascular risk and cognitive performance: A population-based cross-sectional study (NEDICES2-RISK)
Source: PLoS One. 2026 Mar 25;21(3):e0345086. doi: 10.1371/journal.pone.0345086 (PMC13016341; doi:10.1371/journal.pone.0345086)
Supplement: S11 Table — Comparison between participants with the worst score in the TMTA-2 errors test and the rest. (PDF) [file pone.0345086.s012.pdf]

S11 Table. Baseline characteristics of the sample and cardiovascular risk. Comparison between participants with the worst score in the TMTA-Errors 2 and the rest.

	Women				Men			
	**≥P75 (n=149)**	**<P75 (n=353)**	**Overall (N=502)**	***p***	**≥P75 (n=103)**	**<P75 (n=343)**	**Overall (N=446)**	***p***
**Age[1]**	69.0 [64.0–72.0]	67.0 [62.0–71.0]	67.0 [62.0–71.0]	0.006[a]	70.0 [65.0–73.0]	65.0 [61.0–70.5]	67.0 [62.0–71.0]	<0.001[a]
**Education level[2]**								
No education-Primary	101 (68.2)	227 (65.0)	328 (66.0)	0.558[b]	72 (71.3)	179 (52.8)	251 (57.0)	0.001[b]
Secondary-Superior	47 (31.8)	122 (35.0)	169 (34.0)		29 (28.7)	160 (47.2)	189 (43.0)	
**Smoking[2]**								
Non-smoker	102 (69.4)	223 (63.7)	325 (65.4)	0.479[b]	26 (25.7)	87 (25.4)	113 (25.5)	0.896[b]
Smoker	16 (10.9)	45 (12.9)	61 (12.3)		18 (17.8)	55 (16.0)	73 (16.4)	
Ex-smoker	29 (19.7)	82 (23.4)	111 (22.3)		57 (56.4)	201 (58.6)	258 (58.1)	
**Sedentary lifestyle[2]**	114 (76.5)	223 (63.7)	337 (67.5)	0.007[b]	69 (67.6)	210 (61.8)	279 (63.1)	0.336[b]
**Hypertension[2]**	64 (43.0)	167 (47.3)	231 (46.0)	0.426[b]	55 (53.4)	165 (48.1)	220 (49.3)	0.407[b]
**Diabetes Mellitus[2]**	22 (14.8)	45 (12.7)	67 (13.3)	0.643[b]	33 (32.0)	81 (23.6)	114 (25.6)	0.112[b]
**Dyslipidemia[2]**	78 (52.3)	181 (51.3)	259 (51.6)	0.903[b]	50 (48.5)	180 (52.5)	230 (51.6)	0.556[b]
**Atrial fibrillation[2]**	7 (4.7)	6 (1.7)	13 (2.6)	0.104[b]	7 (6.8)	27 (7.9)	34 (7.6)	0.882[b]
**Depression[2]**	33 (22.1)	59 (16.7)	92 (18.3)	0.190[b]	10 (9.7)	24 (7.0)	34 (7.6)	0.485[b]
**CNS treatment[1]**	55 (36.9)	96 (27.2)	151 (30.1)	0.039[b]	20 (19.4)	59 (17.2)	79 (17.7)	0.712[b]
**BMI[1]**	27.9 [24.5–30.4]	27.3 [25.0–30.7]	27.6 [24.8–30.6]	0.923[a]	28.6 [27.0–30.9]	28.7 [26.5–30.7]	28.7 [26.6–30.8]	0.957[a]
**SBP[1]**	129.0 [120.0–140.0]	130.0 [120.0–140.0]	130.0 [120.0–140.0]	0.629[a]	130.0 [121.0–140.0]	132.0 [120.0–142.0]	132.0 [120.0–141.0]	0.560[a]
**DBP[1]**	75.0 [70.0–80.0]	75.0 [70.0–80.0]	75.0 [70.0–80.0]	0.263[a]	75.0 [70.0–80.0]	78.0 [70.0–85.0]	77.0 [70.0–85.0]	0.081[a]
**Total cholesterol[1]**	200.0 [174.0–221.0]	211.0 [185.0–232.3]	208.0 [182.0–231.0]	0.002[a]	183.0 [149.0–210.0]	186.0 [163.0–212.0]	186.0 [160.0–212.0]	0.208[a]
**HDL–c[1]**	57.5 [48.0–70.3]	56.0 [49.0–66.0]	57.0 [49.0–67.0]	0.335[a]	49.0 [40.0–58.0]	48.0 [40.0–56.0]	48.0 [40.0–56.5]	0.417[a]
**REGICOR[2]**								
Low CVR	108 (84.4)	247 (78.4)	355 (80.1)	0.189[c]	36 (43.9)	111 (42.7)	147 (43.0)	0.570[b]
Moderate CVR	18 (14.1)	65 (20.6)	83 (18.7)		32 (39.0)	115 (44.2)	147 (43.0)	
High CVR	2 (1.6)	3 (1.0)	5 (1.1)		14 (17.1)	34 (13.1)	48 (14.0)	
**FRESCO[2]**								
Low CVR	53 (61.6)	129 (60.3)	182 (60.7)	0.877[b]	13 (25.0)	55 (28.1)	68 (27.4)	0.175[b]
Moderate CVR	28 (32.6)	69 (32.2)	97 (32.3)		19 (36.5)	91 (46.4)	110 (44.4)	
High CVR	5 (5.8)	16 (7.5)	21 (7.0)		20 (38.5)	50 (25.5)	70 (28.2)	

TMTA: Trail making test series A (seconds); BMI: Body mass index; SBP: Systolic blood pressure (mmHg); DBP: Diastolic blood pressure (mmHg); HDL-c: High Density Lipoprotein cholesterol; CNS treatment: treatments that modulate the central nervous system; CVR: Cardiovascular risk. 1: median [Q1–Q3]; 2: n (%); a: Mann-Whitney U test; b: Chi-squared test; c: Fisher's test.
