# Supplementary material for: Cardiovascular risk and cognitive performance: A population-based cross-sectional study (NEDICES2-RISK)
Source: PLoS One. 2026 Mar 25;21(3):e0345086. doi: 10.1371/journal.pone.0345086 (PMC13016341; doi:10.1371/journal.pone.0345086)
Supplement: S12 Table — (PDF) [file pone.0345086.s013.pdf]

S12 Table. Effect of cardiovascular risk, measured with the REGICOR and FRESCO equations, on cognitive performance in women.

	Neuropsychological test	Unadjusted			Adjusted[a]		
		OR	CI95%	*p*	OR	CI95%	*p*
REGICOR	**≤P25 of MMSE-37**						
	Low CVR vs Moderate CVR	1.34	0.81–2.18	0.244	1.39	0.81–2.36	0.232
	Low CVR vs High CVR	1.38	0.18–8.43	0.727	1.01	0.12–7.18	0.993
	**≤P25 of Immediate Memory**						
	Low CVR vs Moderate CVR	1.47	0.90–2.41	0.123	1.60	0.95–2.67	0.073
	Low CVR vs High CVR	0.56	0.03–3.83	0.604	0.79	0.04–5.73	0.836
	**≤P25 of Delayed Recall**						
	Low CVR vs Moderate CVR	0.87	0.52–1.44	0.599	0.87	0.51–1.47	0.611
	Low CVR vs High CVR	0.48	0.02–3.27	0.511	0.58	0.03–4.29	0.641
	**≤P25 of Word Accentuation**						
	Low CVR vs Moderate CVR	1.48	0.86–2.48	0.145	1.49	0.82–2.68	0.186
	Low CVR vs High CVR	2.16	0.28–13.20	0.403	1.25	0.15–9.69	0.826
	**≤P25 of Verbal fluency**						
	Low CVR vs Moderate CVR	0.97	0.57–1.62	0.918	0.96	0.55–1.63	0.873
	Low CVR vs High CVR	3.44	0.56–26.40	0.179	2.62	0.39–21.80	0.321
	**<P25 of Clock Drawing**						
	Low CVR vs Moderate CVR	1.43	0.82–2.46	0.201	1.50	0.83–2.69	0.170
	Low CVR vs High CVR	–	–	–	–	–	–
	**≥P75 of TMTA–1**						
	Low CVR vs Moderate CVR	0.97	0.54–1.68	0.904	0.93	0.50–1.68	0.823
	Low CVR vs High CVR	4.80	0.78–36.90	0.089	4.06	0.58–36.80	0.166
	**≥P75 of TMTA–2**						
	Low CVR vs Moderate CVR	1.39	0.80–2.35	0.229	1.41	0.77–2.54	0.252
	Low CVR vs High CVR	5.04	0.82–38.7	0.079	4.11	0.52–44.10	0.196
	**≥P75 of TMTA-Errors 1**						
	Low CVR vs Moderate CVR	0.88	0.50–1.49	0.635	0.80	0.45–1.40	0.453
	Low CVR vs High CVR	0.65	0.03–4.44	0.698	0.58	0.03–4.30	0.639
	**≥P75 of TMTA-Errors 2**						
	Low CVR vs Moderate CVR	0.63	0.35–1.10	0.116	0.61	0.33–1.08	0.101
	Low CVR vs High CVR	1.52	0.20–9.33	0.647	1.69	0.21–11.20	0.583
FRESCO	**≤P25 of MMSE-37**						
	Low CVR vs Moderate CVR	1.74	1.03–2.94	0.036	1.67	0.95–2.95	0.076
	Low CVR vs High CVR	2.50	0.99–6.31	0.050	2.08	0.76–5.70	0.151
	**≤P25 of Immediate Memory**						
	Low CVR vs Moderate CVR	1.31	0.78–2.18	0.301	1.34	0.78–2.28	0.282
	Low CVR vs High CVR	4.13	1.63–11.40	0.004	4.77	1.81–13.80	0.002*
	**≤P25 of Delayed Recall**						
	Low CVR vs Moderate CVR	0.96	0.57–1.60	0.881	0.99	0.57–1.69	0.966
	Low CVR vs High CVR	2.81	1.13–7.44	0.029	2.86	1.09–7.91	0.035*
	**≤P25 of Word Accentuation**						
	Low CVR vs Moderate CVR	1.48	0.84–2.59	0.168	1.29	0.69–2.39	0.426
	Low CVR vs High CVR	3.82	1.51–9.82	0.005	2.83	0.98–8.45	0.056
	**≤P25 of Verbal fluency**						
	Low CVR vs Moderate CVR	0.94	0.55–1.57	0.809	0.75	0.42–1.30	0.307
	Low CVR vs High CVR	1.73	0.69–4.33	0.236	1.42	0.54–3.72	0.474
	**≤P25 of Clock Drawing**						
	Low CVR vs Moderate CVR	2.35	1.29–4.28	0.005	2.08	1.08–4.02	0.029*
	Low CVR vs High CVR	3.62	1.36–9.81	0.010	3.23	1.07–10.10	0.039*
	**≥P75 of TMTA–1**						
	Low CVR vs Moderate CVR	3.21	1.82–5.72	<0.001	2.99	1.62–5.58	<0.001*
	Low CVR vs High CVR	6.94	2.70–18.50	<0.001	6.92	2.43–20.90	<0.001*
	**≥P75 of TMTA–2**						
	Low CVR vs Moderate CVR	2.22	1.24–3.99	0.007	2.01	1.06–3.83	0.032*
	Low CVR vs High CVR	4.52	1.74–11.70	0.002	3.89	1.34–11.50	0.012*
	**>P75 of TMTA-Errors 1**						
	Low CVR vs Moderate CVR	1.52	0.88–2.62	0.134	1.47	0.82–2.63	0.194
	Low CVR vs High CVR	1.99	0.74–5.05	0.154	1.49	0.53–3.94	0.431
	**≥P75 of TMTA-Errors 2**						
	Low CVR vs Moderate CVR	0.99	0.57–1.69	0.964	0.89	0.49–1.57	0.678
	Low CVR vs High CVR	0.76	0.24–2.05	0.611	0.61	0.18–1.71	0.373

CVR: Cardiovascular risk; MMSE–37: Minimental State Examination 37-item version; TMTA: Trail Making Test series A (seconds); CI95%: 95% confidence interval. a: education level, sedentary lifestyle, obesity, atrial fibrillation, depression, treatment affecting the central nervous system; *p<0.05.
