# Supplementary material for: Cardiovascular risk and cognitive performance: A population-based cross-sectional study (NEDICES2-RISK)
Source: PLoS One. 2026 Mar 25;21(3):e0345086. doi: 10.1371/journal.pone.0345086 (PMC13016341; doi:10.1371/journal.pone.0345086)
Supplement: S13 Table — (PDF) [file pone.0345086.s014.pdf]

S13 Table. Effect of cardiovascular risk, measured with the REGICOR and FRESCO equations, on cognitive performance in men.

	Neuropsychological test	Unadjusted			Adjusted[a]		
		OR	CI95%	p	OR	CI95%	p
REGICOR	**≤P25 of MMSE-37**						
	Low CVR vs Moderate CVR	1.45	0.84–2.56	0.188	1.20	0.65–2.23	0.571
	Low CVR vs High CVR	2.94	1.43–6.00	0.003	2.41	1.08–5.36	0.031*
	**≤P25 of Immediate Memory**						
	Low CVR vs Moderate CVR	0.68	0.43–1.09	0.109	0.72	0.45–1.17	0.185
	Low CVR vs High CVR	0.98	0.51–1.89	0.959	0.96	0.49–1.90	0.917
	**≤P25 of Delayed Recall**						
	Low CVR vs Moderate CVR	0.77	0.49–1.23	0.278	0.78	0.48–1.27	0.325
	Low CVR vs High CVR	0.72	0.36–1.40	0.340	0.65	0.31–1.32	0.242
	**≤P25 of Word Accentuation**						
	Low CVR vs Moderate CVR	1.91	1.12–3.30	0.019	1.65	0.90–3.06	0.105
	Low CVR vs High CVR	3.51	1.74–7.11	<0.001	2.90	1.28–6.67	0.011*
	**≤P25 of Verbal fluency**						
	Low CVR vs Moderate CVR	1.61	0.93–2.83	0.089	1.56	0.87–2.82	0.138
	Low CVR vs High CVR	1.85	0.86–3.87	0.109	1.50	0.66–3.31	0.322
	**≤P25 of Clock Drawing**						
	Low CVR vs Moderate CVR	1.16	0.67–2.04	0.599	0.87	0.47–1.61	0.658
	Low CVR vs High CVR	1.54	0.73–3.17	0.248	0.93	0.40–2.11	0.861
	**≥P75 of TMTA–1**						
	Low CVR vs Moderate CVR	2.76	1.50–5.27	0.001	2.21	1.15–4.41	0.020*
	Low CVR vs High CVR	4.19	1.92–9.21	<0.001	3.68	1.57–8.71	0.003*
	**≥P75 of TMTA–2**						
	Low CVR vs Moderate CVR	1.98	1.11–3.60	0.022	1.62	0.87–3.09	0.134
	Low CVR vs High CVR	3.12	1.47–6.58	0.003	2.82	1.24–6.41	0.013*
	**≥P75 of TMTA-Errors 1**						
	Low CVR vs Moderate CVR	0.65	0.36–1.17	0.153	0.60	0.32–1.11	0.107
	Low CVR vs High CVR	1.42	0.67–2.93	0.346	1.39	0.63–2.97	0.409
	**≥P75 of TMTA-Errors 2**						
	Low CVR vs Moderate CVR	0.86	0.50–1.48	0.580	0.72	0.41–1.28	0.267
	Low CVR vs High CVR	1.27	0.60–2.59	0.520	0.92	0.41–1.98	0.837
FRESCO	**≤P25 of MMSE-37**						
	Low CVR vs Moderate CVR	2.35	1.10–5.39	0.033	2.99	1.19–8.27	0.025*
	Low CVR vs High CVR	3.21	1.44–7.61	0.006	2.32	0.87–6.61	0.101
	**≤P25 of Immediate Memory**						
	Low CVR vs Moderate CVR	1.35	0.72–2.57	0.346	1.65	0.85–3.27	0.145
	Low CVR vs High CVR	2.08	1.06–4.15	0.036	2.23	1.07–4.70	0.033*
	**≤P25 of Delayed Recall**						
	Low CVR vs Moderate CVR	0.76	0.41–1.41	0.378	0.83	0.43–1.64	0.593
	Low CVR vs High CVR	1.46	0.75–2.85	0.264	1.55	0.74–3.25	0.245
	**≤P25 of Word Accentuation**						
	Low CVR vs Moderate CVR	2.24	1.07–4.98	0.039	2.09	0.92–4.99	0.086
	Low CVR vs High CVR	4.00	1.85–9.19	<0.001	2.99	1.25–7.53	0.016*
	**≤P25 of Verbal fluency**						
	Low CVR vs Moderate CVR	1.29	0.61–2.86	0.520	1.50	0.65–3.66	0.351
	Low CVR vs High CVR	1.64	0.74–3.79	0.232	1.40	0.57–3.59	0.469
	**≤P25 of Clock Drawing**						
	Low CVR vs Moderate CVR	1.09	0.53–2.28	0.822	0.94	0.42–2.11	0.883
	Low CVR vs High CVR	1.90	0.89–4.17	0.101	1.33	0.56–3.19	0.517
	**≥P75 of TMTA–1**						
	Low CVR vs Moderate CVR	2.32	1.02–5.81	0.055	2.01	0.82–5.42	0.143
	Low CVR vs High CVR	5.00	2.16–12.7	<0.001	3.72	1.46–10.3	0.008*
	**≥P75 of TMTA–2**						
	Low CVR vs Moderate CVR	1.59	0.76–3.50	0.228	1.43	0.63–3.40	0.399
	Low CVR vs High CVR	2.65	1.22–6.02	0.016	2.15	0.90–5.36	0.090
	**≥P75 of TMTA-Errors 1**						
	Low CVR vs Moderate CVR	0.95	0.45–2.06	0.901	1.16	0.51–2.75	0.736
	Low CVR vs High CVR	1.57	0.72–3.51	0.257	1.66	0.68–4.17	0.269
	**≥P75 of TMTA-Errors 2**						
	Low CVR vs Moderate CVR	0.88	0.41–1.96	0.756	0.85	0.37–2.02	0.708
	Low CVR vs High CVR	1.69	0.77–3.82	0.195	1.23	0.51–3.02	0.649

CVR: Cardiovascular risk; MMSE–37: Minimental State Examination 37-item version; TMTA: Trail Making Test series A (seconds); CI95%: 95% confidence interval. a: education level, sedentary lifestyle, obesity, atrial fibrillation, depression, treatment affecting the central nervous system; *p<0.05.
