# Supplementary material for: Cardiovascular risk and cognitive performance: A population-based cross-sectional study (NEDICES2-RISK)
Source: PLoS One. 2026 Mar 25;21(3):e0345086. doi: 10.1371/journal.pone.0345086 (PMC13016341; doi:10.1371/journal.pone.0345086)
Supplement: Attachment — Submitted filename: Response to Reviewers.docx [file pone.0345086.s016.docx]

**Response to Reviewers**

We would like to sincerely thank the Editor and the Reviewers for their thorough evaluation of our manuscript and for the constructive and insightful comments provided. We greatly appreciate the time and expertise invested in reviewing our work. We have carefully considered each observation and have revised the manuscript accordingly. Below, we provide a point-by-point response detailing the changes made and clarifying the issues raised.

In addition, we have incorporated the other modifications previously communicated to the Editor in August, which we were asked to introduce once the reviewers´ feedback became available. These additional modifications stem from the fact that, during the review period, we continued to perform additional analyses of the dataset in preparation for future publications. During this work, we identified that some subjects had been incorrectly classified with respect to their cardiovascular risk and we were also able to include the data from 4 additional participants in the revised analysis. While these corrections does not alter the main findings or overall conclusions of the previously submitted manuscript, we consider it important to update the text to maintain the highest level of accuracy and scientific rigor.

Changes made in response to the reviewers´comments are highlighted in yellow, while the additional modifications we propose to include are marked in blue in the revised manuscript.

We hope that the revised versión meets the expectations of the Editor and the Reviewers and we remain at your disposal foro any further clarification or adjustments needed.

**Reviewer #1: First of all I should say Authors did really valuable work on comparing CVR and cognitive performance, I just give some recommendation to improve their work:**

**How authors follow potential confounders in their studies?**

Thank you very much for your appreciation. To address the possible influence of confounding variables, we developed a Directed Acyclic Graph (DAG) representing the causal relationships between the study variables. This diagram allowed us to identify potential confounding pathways between exposure (cardiovascular risk) and outcome (cognitive performance) and determine the variables that should be adjusted in statistical analyses to obtain unbiased estimates of the causal effect.

We attach the image that has been included in the supplements section of the manuscript: Figure S1. Directed Acyclic Graph (DAG) created with DAGitty v3.1 to identify paths of confusion between exposure (cardiovascular risk) and outcome (cognitive performance).

In the methodology section of the manuscript, we have included the following sentence: A Directed Acyclic Graph (DAG) was constructed using DAGitty v3.1 to identify possible paths of confusion between exposure (cardiovascular risk) and outcome (cognitive performance).

The final criteria for the inclusion of confounding variables were based on (1) the development of the DAG, (2) the consideration of clinical variables relevant to our study objective, and (3) the identification of those that showed statistically significant differences.

Some potential covariates were not included in the model because they form part of the calculation of the risk value itself in the REGICOR and FRESCO tools, which could generate collinearity, affecting the stability of the model and the interpretation of the estimates. REGICOR includes the following variables in the risk calculation: age, sex, smoking, systolic and diastolic blood pressure, total cholesterol, HDL cholesterol, and diabetes. FRESCO, meanwhile, incorporates age, sex, smoking, systolic blood pressure, total cholesterol, HDL cholesterol, diabetes, and lipid-lowering treatment.

We have included the following sentence in the manuscript to clarify the above comment: Some potential covariates were not included in the model, as they are integrated into the risk value calculation in the REGICOR and FRESCO tools, and their additional inclusion could induce collinearity.

With all this in mind, we adjusted the generalised linear models for the following confounding variables: educational level (no education or primary education vs. secondary or superior education), obesity (presence if BMI ≥30 or absence), AF (presence or absence), depression (presence or absence), physical exercise (active or inactive), and treatment modulating the CNS (anxiolytics, neuroleptics, antidepressants, and antiepileptic drugs).

**How authors address recall bias of cohort study? Because Data extract from cohort study.**

We appreciate the reviewer´s concern regarding potential recall bias in our cohort study. In this regard, we would like to clarify the following:

- Objective clinical measurements: blood pressure, weight and height were obtained through direct measurement by trained clinical personnel. Therefore, these variables were no subject to participant recall and were not affected by recall bias.
- Medical history and comorbidities: information on participants´ medical conditions was collected by the study physicians during clinical encounters and verified against medical records. These data were not self-reported by participants.
- Neuropsychological assessmentes: all neuropsychological tests were administered in person during structured interviews conducted by qualified staff. The results reflect direct performance-based measures rather than recalled information.
- Missing data: any missing clinical information was retrieved from participants´ electronic medical records, ensuring accuracy and continuity of data without relying on participant memory.

For this reasons, recall bias is unlikely to have influenced our findings, as key variables were either directly measured, clinically assessed or extracted from medical records rather than self-reported.

We thank the reviewer for their insightful comments. Their observations made us realize that the manuscript did not explicitly describe how the study data were obtained. We will therefore include the following two paragraphs in the revised versión clarifying the data Collection procedures.

Clinical data, including blood pressure, weight and height, were obtained through direct measurements performed by trained clinical staff during study visits. Information on participants´ medical history and comorbidities was collected by the study physicians and verified using medical records. When necessary, missing clinical information was completed through review of the electronic medical records.

These neuropsychological tests were administered in person during structured interviews conducted by qualified personnel.

**Explain how the study size was arrived at?**

The study population was drawn from the NEDICES-2 (Neurological Disorders in Central Spain) cohort study. Patient recruitment took place in the primary care setting within the Spanish National Health System in the regions of Ávila, Madrid, Salamanca, and Segovia, Spain. The population in the study NEDICES2 originated from the list

of users (social security card holders) of the participating doctors in the included healthcare centers, who were then selected following randomized sampling of patients ≥ 55 years-old stratified by gender and age (5-year intervals).

A specific sub-cohort, NEDICES2-RISK, was extracted from the original cohort study, which included all subjects in the NEDICES-2 cohort aged 55–75 years without dementia who underwent neuropsychological assessment. The sample size was determined by the number of participants who met these inclusion criteria.

**Explain how missing data were addressed?**

Participants with incomplete information on key variables for calculating cardiovascular risk were excluded from the analysis (complete case analysis). The proportion of missing data was low (0.52 %), only 5 participants, so it is unlikely that this procedure introduced significant bias.

In the case of neuropsychological tests, it was not possible to complete the full battery of tests in all patients, so an analysis of each test was performed with the patients who had completed it, excluding the rest. The number of patients analysed in each test is specified in the supplements (S2-S11).

**Indicate number of participants with missing data for each variable of interest.**

Attached here are the patients included in each CVR analysis and those who were able to complete each neuropsychological test:

| Risk functions | Included | Excluded from the calculation in one of the risk functions | Excluded from the calculation in both |
| --- | --- | --- | --- |
| Analysed with REGICOR | 795 | 68 (age 75) | 94 with cardiovascular events  5 with incomplete data |
| Analysed with FRESCO | 555 | 308 (with lipid-lowering treatment) |  |

| TEST |  | |
| --- | --- | --- |
|  | **Includad** | **NA** |
| MMSE-37 | 962 | 0 |
| Immediate Recall test | 960 | 2 |
| Delayed Recall test | 960 | 2 |
| Word Accentuation test | 944 | 18 |
| Verbal Fluency test | 958 | 4 |
| Clock Drawing test | 760 | 202 |
| TMTA-1 | 949 | 13 |
| TMTA-2 | 948 | 14 |
| TMTA-Errors 1 | 945 | 17 |
| TMTA-Errors 2 | 943 | 19 |

**Reviewer #2: The introduction is comprehensive and well-referenced, giving a clear overview of the global and national context of dementia and cardiovascular disease. It correctly emphasizes the importance of modifiable risk factors and the need to study these relationships in Mediterranean populations.**

**That said, the section could be more focused. Several paragraphs repeat well-known information on dementia trends and cardiovascular epidemiology. The rationale for using the REGICOR and FRESCO equations in relation to cognition appears late and could be highlighted earlier**

We thank the reviewer for this helpful observation. In response, we have streamlined the introduction by removing repetitive sentences related to dementia trends and cardiovascular epidemiology, ensuring that only the most essential background information is retained.

**The authors convincingly justify the inclusion of sex-stratified analyses, given known biological and lifestyle differences. However, the introduction would benefit from a more explicit hypothesis and a brief conceptual framework linking cardiovascular pathology (e.g., microvascular damage, inflammation) to specific cognitive domains such as memory and processing speed.**

We appreciate the reviewer´s valuable comment regarding the need for a more explicit hypothesis with a brief conceptual framework linking cardiovascular pathology to specific cognitive domains, including memory and processing speed. We agree that the evidence supporting these mechanism warrants clearer emphasis. Accordingly, we have revised the introduction to articulate the study hypothesis more explicity and to incorporate a concise conceptual framework supported by the appropiate references. We trust that these additions strengthen the manuscript and address the reviewer´s recommendation.

The added paragraph was the following:

Although the pathophysiological mechanisms of dementia remain unclear, several studies in general population samples have shown that microvascular dysfunction (assessed through biomarkers, imaging of small vessel disease or arterial stiffness) is associated with poorer performance in specific domains such as memory and processing speed.

**Selection bias: Excluding participants with prior cardiovascular events or those taking lipid-lowering drugs (for FRESCO) may skew the sample toward healthier individuals, underestimating the true relationship between CVR and cognition.**

Thank you very much for your contribution, which we consider very relevant and interesting. With regard to possible selection bias, participants with previous cardiovascular events were excluded, as the aim of our study was to assess how cardiovascular risk, estimated using the REGICOR and FRESCO tools, influences cognitive performance. These tools calculate the absolute 10-year risk of a cardiovascular event, so they cannot be applied to individuals who have already suffered an event.

Additionally, the FRESCO tool specifically excludes patients who are taking lipid-lowering treatment, as these drugs alter lipid levels and could skew the cardiovascular risk estimate. This exclusion is part of the risk calculator's own criteria.

However, we also find it interesting to analyse cognitive performance in patients who have already suffered a cardiovascular event in order to broaden our understanding of the impact of cardiovascular events on cognitive function, and we have therefore included this as a proposal for future complementary lines of research.

REGICOR and FRESCO tools calculate the absolute 10-year risk of experiencing a cardiovascular event, so they cannot be applied to individuals who have already suffered an event. It would be interesting to analyse cognitive performance in patients who have already suffered a cardiovascular event in order to broaden our understanding of the impact of cardiovascular events on cognitive function, and we therefore include this as a proposal for future complementary lines of research.

**Outcome measurement: Although the neuropsychological battery is broad, the brief nature of some tests (e.g., 6-item recall, short fluency task) may reduce sensitivity to subtle cognitive differences.**

We appreciate the reviewer´s observation regarding the potential reduction in sensitivity when using brief cognitive tests. We agree that shorter assessments may have limitations compared with more comprehensive neuropsuchological batteries.

Despite the low sensitivity of some of these tests, they have been added to the battery to provide a more comprehensive view of the different cognitive aspects. We use a total of seven different tests that take into account six different cognitive domains (global cognition, memory, premorbid intelligence, verbal fluency, visuoconstruction, attention, and psychomotor skills). Furthermore, we believe it is interesting to observe the performance of the brief tests because they are the most suitable for use in clinical practice.

**Statistical approach: Dichotomizing cognitive scores into quartiles (≤25th percentile vs. >25th) leads to a loss of information and power. Modeling continuous outcomes could have provided more nuanced results. Additionally, the use of multiple comparisons without correction raises the possibility of false-positive findings.**

We appreciate the reviewer´s comment and fully agree that, as noted, dichotomizing continuous outcomes can lead to a loss of information and statistical power. Nonetheless, we selected this approach to facilitate clinical interpretability and consistency with prior studies in the field.

The decision to divide cognitive scores into quartiles (≤25th percentile vs. >25th) was made because, at the start of the study, participants had very similar cognitive scores, resulting in a non-normal distribution. Attached is a table summarising the results of the normality tests, as well as the corresponding histograms for each neuropsychological test.

Below is the table with the p-values obtained after applying the Anderson-Darling test. When the p-value is extremely small for the Anderson-Darling test, R shows it in scientific notation as 3.7e-24.

| **Neuropsichological tests** | **Anderson-Darling Test** | **Kolmogorov-Smirvov Test** |
| --- | --- | --- |
| MMSE-37 | 3.7e-24 | 1.69e-21 |
| Immediate Recall test | 3.7e-24 | 8.76e-37 |
| Delayed Recall test | 3.7e-24 | 2.25e-36 |
| Word Accentuation test | 3.7e-24 | 1.55e-17 |
| Verbal Fluency test | 5.71e-13 | 7.23e-06 |
| Clock Drawing test | 3.7e-24 | 6.59e-107 |
| TMTA-1 | 3.7e-24 | 8.75e-14 |
| TMTA-2 | 3.7e-24 | 2.00e-22 |
| TMTA-Errors 1 | 3.7e-24 | 1.81e-118 |
| TMTA-Errors 2 | 3.7e-24 | 4.33e-74 |

All tests obtained a p-value lower than 0.05, therefore, the null hypothesis of normality is rejected, concluding that the data do not follow a normal distribution.

Quartile stratification allows for more accurate identification of participants with lower cognitive performance compared to those with higher performance, facilitating comparisons between groups and offering results that are more easily interpretable from a clinical point of view. Conversely, when modelling data continuously, this low variability between values could hinder the detection of significant associations and complicate the interpretation of results.

Thank you for your comment. We have included the following explanation of the reason for the quartile division in the methodology section.

The decision to divide cognitive scores into quartiles (≤25th percentile vs. >25th) was made because, at the start of the study, participants had very similar cognitive scores, resulting in a non-normal distribution and because quartile stratification allows for more accurate identification of participants with lower cognitive performance compared to those with higher performance, facilitating comparisons between groups and offering results that are more easily interpretable from a clinical point of view.

**Residual confounding: Important variables such as diet, alcohol intake, sleep, and socioeconomic status were not considered, though all are known to influence both cardiovascular and cognitive health.**

We fully agree with the reviewer that variables such as diet, alcohol intake, sleep and socioeconomic status can influence both cardiovascular ande cognitive health. However, dieta and socioeconomic status were not collected within our cohort and therefore could not be incorporated into the analyses.

The sleep data available in the cohort did not meet the quality standards required for robust analysis, as they were not collected systematically nor with validated instruments and therefore could not be reliably incorporated into the models. Regarding alcohol consumption, this variable was not included because it did not materially affect the original odds ratios, and when analysed separately, its associations were non-significant. For these reasons, neither sleep nor alcohol consumption were retained as confounding variables in the final models.

**The discussion section appropriately interprets the main results within the context of existing literature. The authors correctly note that their findings align with prior studies showing that higher cardiovascular burden correlates with poorer cognitive function. They also highlight that memory and processing speed were the domains most affected—consistent with vascular contributions to cognitive decline.** **Nevertheless, the discussion could be more critical in tone. Several points could be strengthened**

**The possibility of reverse causation should be explicitly addressed**

We appreciate the suggestion and we have now explicitly addressed this issue in the manuscript. The following text has been added to the revised version.

An additional consideration is the potential for reverse causation, but epidemiological evidence supports a direct causal relationship between cardiovascular risk factors and cognitive impairment. Longitudinal cohorts have shown that increased cardiovascular risk in middle age is associated with accelerated cognitive decline and increased risk of dementia[75,76]. Mendelian randomisation studies reinforce this relationship. A genetic predisposition to hypertension and dyslipidaemia is linked to an increased risk of Alzheimer's disease, suggesting a causal effect [77–79]. However, some findings may reflect reverse causality. In preclinical stages of dementia, changes in blood pressure, weight and cholesterol are observed, indicating that incipient neurodegeneration may modify cardiovascular parameters prior to diagnosis [80,81].

**Mechanistic explanations (e.g., cerebral microvascular damage, systemic inflammation, impaired perfusion) are only briefly mentioned and could be elaborated**

In accordance with the reviewer's suggestion, we have added an explanation of the mechanisms of neurodegenerative processes to the discussion.

Dementia arises from the interaction between vascular dysfunction and neurodegenerative processes. Cerebrovascular dysfunction, especially in the microvasculature, causes cerebral hypoperfusion, impaired blood flow autoregulation, and structural damage to arterioles and capillaries. This generates oxidative stress, hypoxia, and mitochondrial dysfunction, affecting neuronal energy metabolism and causing synaptic damage.

The blood-brain barrier becomes permeable, allowing the filtration of toxic molecules and activating neuroinflammation through microglia and astrocytes. These vascular processes not only induce direct neuronal damage, but also enhance the classic mechanisms of dementia such as the accumulation of beta-amyloid and tau. Together, the interaction between vascular alterations, oxidative stress, neuroinflammation, and protein alterations leads to the progressive cognitive decline characteristic of dementia(67).

Our findings linking cardiovascular risk with cognitive performance are consistent with the growing body of evidence supporting the role of vascular pathophysiology in cognitive decline and dementia.

**The observation that smokers and ex-smokers performed better in some tests is intriguing but counterintuitive; this finding deserves cautious interpretation and deeper exploration, as it may reflect cohort or survivor effects rather than a genuine protective association.**

We thank the reviewer for this helpful observation, which prompted us to explore the issue further and to include the additional information presented below.

Given the unexpected results of the association between tobacco use and improved cognitive performance, we analysed possible confounding factors and found that smokers and ex-smokers are younger and have a higher level of education, which would justify better performance as both variables are associated with the same.

|  | **Non-smoker (N=420)** | **Smoker (N=114)** | **Ex-smoker (N=324)** | **Valor-p** |
| --- | --- | --- | --- | --- |
| **Sex^1^** |  |  |  |  |
| Women | 315 (75.0%) | 59 (51.8%) | 106 (32.7%) | <0.001^a^ |
| Men | 105 (25.0%) | 55 (48.2%) | 218 (67.3%) |  |
| **Age^1^** |  |  |  |  |
| Median [Q1-Q3] | 69.00 [64.00-72.00] | 63.00 [60.00-68.00] | 65.00 [61.00-71.00] | <0.001^a^ |
| **Education level^2^** |  |  |  |  |
| No education-Primary | 305 (73.3%) | 56 (50.0%) | 168 (52.2%) | <0.001^b^ |
| Secondary-Superior | 111 (26.7%) | 56 (50.0%) | 154 (47.8%) |  |
| **Physical activity^2^** |  |  |  |  |
| No | 281 (67.2%) | 77 (68.8%) | 192 (59.4%) | 0.054^b^ |
| Yes | 137 (32.8%) | 35 (31.3%) | 131 (40.6%) |  |
| **Hypertension^2^** |  |  |  |  |
| No | 222 (52.9%) | 69 (60.5%) | 177 (54.6%) | 0.345^b^ |
| Yes | 198 (47.1%) | 45 (39.5%) | 147 (45.4%) |  |
| **Diabetes mellitus^2^** |  |  |  |  |
| No | 354 (84.3%) | 92 (80.7%) | 267 (82.4%) | 0.607^b^ |
| Yes | 66 (15.7%) | 22 (19.3%) | 57 (17.6%) |  |
| **Dyslipidemia^2^** |  |  |  |  |
| No | 236 (56.2%) | 58 (50.9%) | 154 (47.5%) | 0.061^b^ |
| Yes | 184 (43.8%) | 56 (49.1%) | 170 (52.5%) |  |
| **Atrial fibrillation^2^** |  |  |  |  |
| No | 408 (97.1%) | 108 (94.7%) | 306 (94.4%) | 0.158^b^ |
| Yes | 12 (2.9%) | 6 (5.3%) | 18 (5.6%) |  |
| **Depression^2^** |  |  |  |  |
| No | 366 (87.1%) | 88 (77.2%) | 284 (87.7%) | 0.014^b^ |
| Yes | 54 (12.9%) | 26 (22.8%) | 40 (12.3%) |  |
| **CNS treatment^2^** |  |  |  |  |
| No | 324 (77.1%) | 78 (68.4%) | 249 (76.9%) | 0.135^b^ |
| Yes | 96 (22.9%) | 36 (31.6%) | 75 (23.1%) |  |
| **BMI^1^** |  |  |  |  |
| Median [Q1-Q3] | 27.98 [25.58-30.86] | 27.23 [24.42-29.81] | 28.38 [25.91-30.56] | 0.032^a^ |

**The authors could also discuss the implications of their findings for preventive strategies in primary care, particularly since REGICOR and FRESCO are tools already used in clinical settings.**

We fully agree with the reviewer´s comment, and we have expanded the applicability section accordingly by incorporating the suggested observations.

Based on our conclusions, and in particular on prospective studies supported by longitudinal designs, the use of these tools, which are already used in clinical practice, especially in primary care, could be extended beyond their traditional applications. Identifying associations between cardiovascular risk and cognitive performance would not only help guide decisions regarding the indication and intensity of cardiovascular risk treatment aimed at preventing cardiovascular events, but would also promote a more comprehensive view of the patient, as it considers cognitive aspects and aims to reduce the incidence of cognitive impairment and dementia and prevent their progression. In this way, these tools, FRESCO and REGICOR may contribute to a more personalized and preventive approach integrating both cardivascular health and cognitive well-being.
