# Supplementary material for: Cardiovascular risk and cognitive performance: A population-based cross-sectional study (NEDICES2-RISK)
Source: PLoS One. 2026 Mar 25;21(3):e0345086. doi: 10.1371/journal.pone.0345086 (PMC13016341; doi:10.1371/journal.pone.0345086)
Supplement: Attachment — Submitted filename: Response_to_Reviewers_auresp_2.docx [file pone.0345086.s017.docx]

Dear Reviewer,

We would like to sincerely thank you for your careful review of our revised manuscript and for your very positive and constructive comments.

We greatly appreciate your recognition of the improvements made to the manuscript in response to the reviewers’ suggestions, as well as your acknowledgment of the additional analyses and clarifications we included beyond what was initially requested. Your assessment that these modifications strengthen the quality, rigor, and balance of the study is particularly encouraging.

We have carefully considered your comments and suggestions. Below, we provide our responses to the specific points you raised and indicate the corresponding revisions made in the manuscript.

We have highlighted in yellow the additional text that we propose to include in the manuscript.

Thank you again for your insightful comments and for helping us improve the quality of our study.

Kind regards,

Ester Tapias

1**. While the authors provide a clear flow chart for how the analytical sample size was arrived at (Figure 1), they should describe this process much clearer in the text. For example, it is not clear that if the initial sample of 962 participants included only people between the ages of 55-74 years, why did they have to exclude a further 68 individuals who were 75 years or older when the REGIDOR score was analysed.**

**In lines 138-141 on page 6, they should also mention the final sample sizes for the two scores. Particularly for the FRESCO score, a very large proportion of participants were excluded due to taking lipid lowering agents, and this needs to be explicitly presented in the text.**

We thank the Editor for this comment and fully agree that the description of the analytical sample selection process should be clarified in the text. Although participants were selected based on being between 55 and 75 years of age at the time of inclusión in the study, this age criterion referred specifically to their age at enrollment. Due to organizational and logistical aspects of the study implementation, including delays related to funding execution and the hiring of evaluators, there was an approximate two-year gap between participant inclusion and the cognitive assessment interviews. As a result, some participants had exceded the upper age limit by the time the REGICOR scores was analyzed, which explains the exclusion of individuals aged 75 years or older at assessment. We have now clarified this process in the revised manuscript to improve transparency and readability.

Based on the expansión of the information in lines 138-141:

The present study included subjects 55–75 years of age without dementia who were part of the NEDICES-2 cohort and had undergone neuropsychological assessment. The resulting sample was comprised of 962 patients that constitute the NEDICES2-RISK cohort. Of these participants, 94 had suffered cardiovascular events (ischemic heart disease, stroke, and/or peripheral artery disease) and were therefore excluded from the analysis, as the CVR functions can only be utilized in the absence of such events [49]. Following the FRESCO guidelines, 308 patients under treatment with lipid-lowering agents were also excluded from the sample whose CVR was estimated using this function [31]. Therefore, for the calculation of cardiovascular risk using the REGICOR score, a total of 795 patients were ultimately analyzed using REGICOR and 555 using FRESCO.

**I`m also wondering whether the differences that we see between the REGIDOR and FRESCO score results are due to these differences in the analytical sample sizes, or due to real differences in the strength of the associations regarding these two scores.**

We thank the Editor for this thoughtful comment regarding the differences observed between the REGICOR and FRESCO results. As described in the Methods section (page 8, lines 166–173), both REGICOR and FRESCO are tools designed to estimate 10-year cardiovascular risk; however, they differ in the outcomes they predict. REGICOR, the Spanish adaptation of the Framingham risk function, estimates the 10-year risk of coronary heart disease events, whereas FRESCO estimates the 10-year risk of global cardiovascular events, including coronary heart disease and stroke. As a result, the FRESCO function classifies a larger proportion of individuals as being at high cardiovascular risk compared with REGICOR, which is known to be more closely aligned with Framingham estimates. Therefore, we think the differences observed between the associations obtained with REGICOR and FRESCO are related to genuine differences in the definition and scope of cardiovascular risk captured by each score.

We have added the following explanatory sentence to the Methods section:The FRESCO function classifies more people as high risk than REGICOR function. [51]

**2. I would also suggest that the main findings as described in lines 425-427 (page 24) should be much more nuanced. It is true that the results indicate significant inverse associations for some of the cognitive function indicators, but there were several non-significant associations as well. Therefore, the blanket statement of both risk scores being associated with poorer cognitive performance is not correct.**

We thank the reviewer for this comment. We agree that the original wording of the main findings could be too general and not sufficiently reflect that not all associations between cardiovascular risk scores and cognitive outcomes reached statistical significance.

To address this concern, we have revised the text in lines 425–427 to provide a more nuanced interpretation of our results. Specifically, we could say that moderate or high cardiovascular risk, as estimated using the REGICOR and FRESCO risk equations, was associated with poorer cognitive performance in the majority of the cognitive tests analyzed, rather than implying a consistent association across all cognitive indicators.

We believe that this modification could represente more accurately our findings while maintaining a clear and concise summary of the main results.

**3. The authors did not really respond to the comment regarding multiple testing. As previous reviewer 2 correctly mentions, when so many associations are examined simultaneously, the issue of multiple testing and possibility of false positive associations may occur. As a minimum, I suggest mentioning this in the Limitations section of the manuscript, or maybe considering the application of Bonferroni correction or similar approach**

We thank the reviewer for pointing out this issue and apologize for not addressing this comment adequately in our previous response.

As noted by the reviewer, when multiple comparisons are performed, there is an increased risk of obtaining statistically significant results by chance alone, leading to a higher probability of type I error (false positives). Following this suggestion, we can explicitly acknowledged this issue by including it as a limitation in the Discussion section of the manuscript:

Finally, we would like to note that the performance of multiple statistical comparisons may increase the likelihood of obtaining statistically significant results by chance alone, thereby raising the risk of Type I errors, which should be taken into account when interpreting the findings.

We believe that incorporating this limitation appropriately addresses the reviewer’s concern regarding multiple testing and provides a more transparent interpretation of our findings.
